# Supplementary material for: The CIP2A-TOPBP1 axis facilitates mitotic DNA repair via MiDAS and MMEJ
Source: Nat Commun. 2025 Nov 27;16:10623. doi: 10.1038/s41467-025-65594-2 (PMC12661054; doi:10.1038/s41467-025-65594-2)
Supplement: Supplementary file 1 — Supplementary Information [file 41467_2025_65594_MOESM1_ESM.pdf]

The CIP2A-TOPBP1 axis facilitates mitotic DNA repair via MiDAS and MMEJ

Peter R Martin, Jadwiga Nieminuszczy, Zuza Kozik, Nihal Jakub, Szymon Kowalski, Maxime Lecot, Julia Vorhauser, Karen A Lane, Alexandra Kanellou, Jörg Mansfeld, Laurence H Pearl, Antony W Oliver, Jessica A Downs, Jyoti Choudhary, Matthew Day, Wojciech Niedzwiedz.

Supplementary Information and Figures

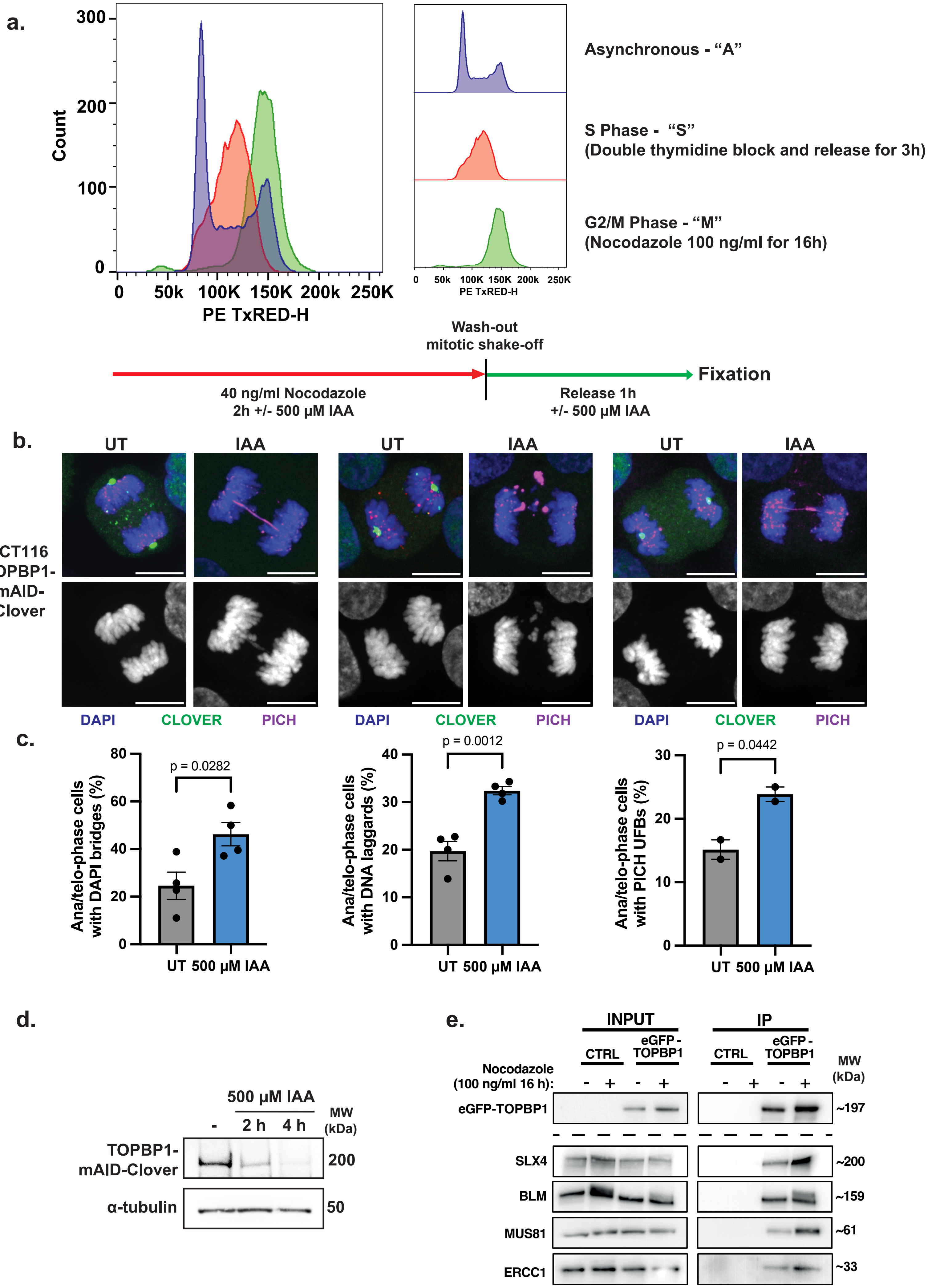
a.
Count
PE TxRED-H
Asynchronous - "A"
S Phase - "S"
(Double thymidine block and release for 3h)
G2/M Phase - "M"
(Nocodazole 100 ng/ml for 16h)
Wash-out
mitotic shake-off
Fixation
40 ng/ml Nocodazole
2h +/- 500 μM IAA
Release 1h
+/- 500 μM IAA
b.
UT
IAA
HCT116
TOPBP1-
mAID-
Clover
DAPI
CLOVER
PICH
c.
Ana/telo-phase cells with DAPI bridges (%)
p = 0.0282
Ana/telo-phase cells with DNA laggards (%)
p = 0.0012
Ana/telo-phase cells with PICH UFBs (%)
p = 0.0442
UT
500 μM IAA
d.
500 μM IAA
-
2 h
4 h
MW (kDa)
TOPBP1-mAID-Clover
200
α-tubulin
50
e.
INPUT
IP
CTRL
eGFP-TOPBP1
Nocodazole (100 ng/ml 16 h):
eGFP-TOPBP1
SLX4
BLM
MUS81
ERCC1
~197
~200
~159
~61
~33

Supplementary Figure 1:
(a) Representative histogram plots of flow-cytometry analysis of propidium iodide stained HEK293TN asynchronous, S-phase synchronised and M-phase synchronised cells, as carried out in experiments described in Figure 1 for Co-IP and mass-spectrometry analysis. (b) Representative images and treatment schematic of HCT116-TOPBP1-mAID-Clover cells treated with or without 500 µM IAA as indicated. (c) Bar plots of anaphase abnormalities after acute TOPBP1 degradation: Percentage of anaphases with DAPI marked chromatin bridges (-: n= 111, +: =125 from four independent experiments); Percentage of anaphase cells with DAPI marked DNA laggards, (-: n= 111, +: =125 from four independent experiments). Percentage of anaphases with PICH marked ultra-fine anaphase bridges (-: n= 40, +: =47 from two independent experiments). Statistical significance in c was determined by two tailed unpaired t-tests. Black dots represent values from each experiment; bars represent mean and error bars indicate SEM. Scale bars equivalent to 10 µm. (d) Western blot showing analysis of degradation of endogenously mAID-Clover tagged TOPBP1 in HCT116-ostir1 cells with or without treatment with 500 µM IAA as indicated. (e) Western blot analysis of BLM, ERCC1, MUS81 and SLX4 interactions with eGFP-TOPBP1 in GFP-TRAP Co-IPs from HEK293TN cells transiently transfected with an eGFP-TOPBP1 WT expression construct followed with or without mitotic synchronisation by 100 ng/ml nocodazole for 18 hours. 1% of input was used for analysis by western blot of input lysate. Source data are provided as a Source Data file.

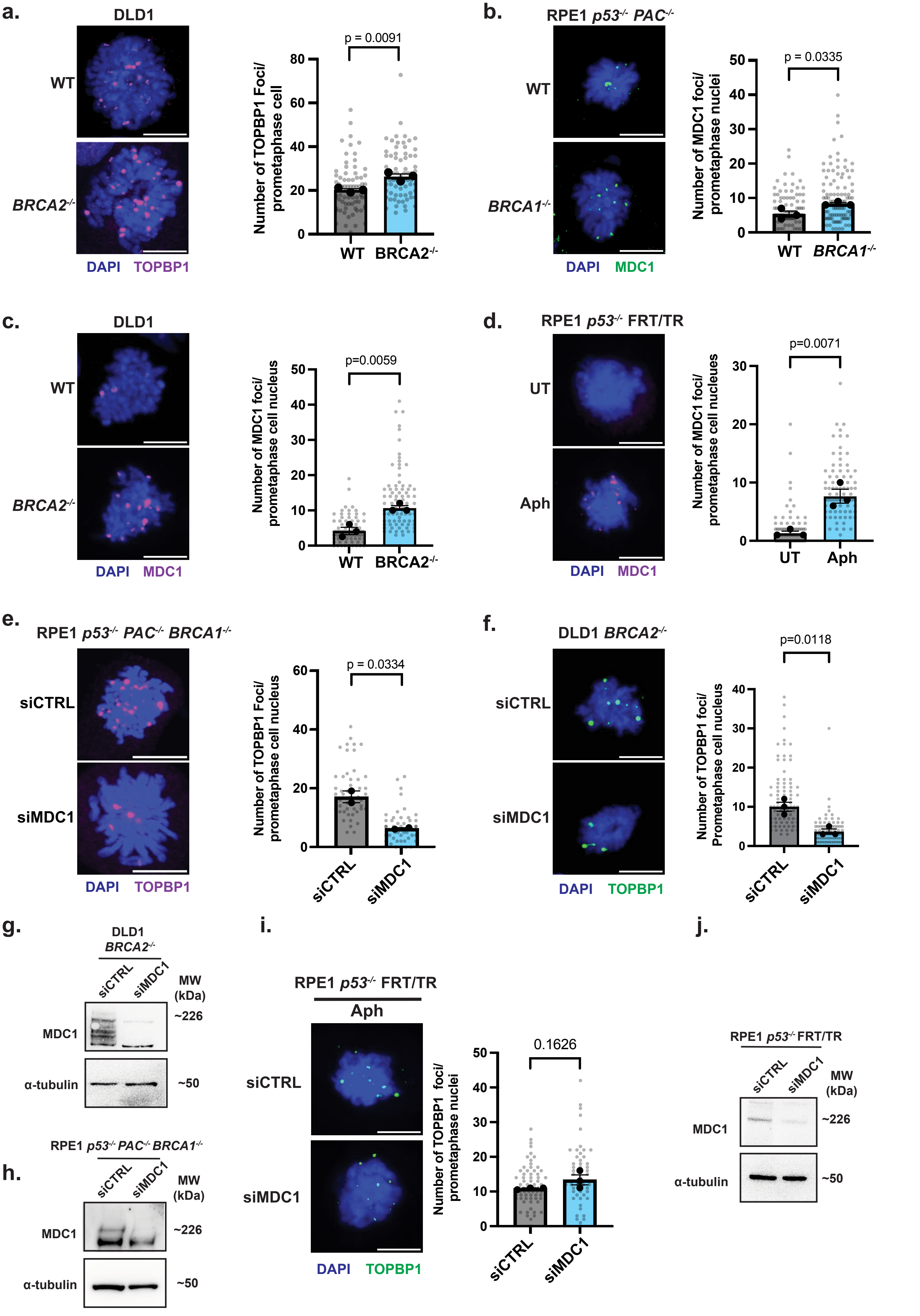
a.
DLD1
WT
BRCA2-/-
DAPI TOPBP1
p = 0.0091
Number of TOPBP1 Foci/ prometaphase cell
0 20 40 60 80
WT BRCA2-/-
b.
RPE1 p53-/- PAC-/-
WT
BRCA1-/-
DAPI MDC1
p = 0.0335
Number of MDC1 foci/ prometaphase nuclei
0 10 20 30 40 50
WT BRCA1-/-
c.
DLD1
WT
BRCA2-/-
DAPI MDC1
p=0.0059
Number of MDC1 foci/ prometaphase cell nucleus
0 10 20 30 40 50
WT BRCA2-/-
d.
RPE1 p53-/- FRT/TR
UT
Aph
DAPI MDC1
p=0.0071
Number of MDC1 foci/ prometaphase cell nucleues
0 10 20 30
UT Aph
e.
RPE1 p53-/- PAC-/- BRCA1-/-
siCTRL
siMDC1
DAPI TOPBP1
p = 0.0334
Number of TOPBP1 Foci/ prometaphase cell nucleus
0 20 40 60
siCTRL siMDC1
f.
DLD1 BRCA2-/-
siCTRL
siMDC1
DAPI TOPBP1
p=0.0118
Number of TOPBP1 foci/ Prometaphase cell nucleus
0 10 20 30 40
siCTRL siMDC1
g.
DLD1 BRCA2-/-
siCTRL siMDC1
MW (kDa)
MDC1 ~226
α-tubulin ~50
h.
RPE1 p53-/- PAC-/- BRCA1-/-
siCTRL siMDC1
MW (kDa)
MDC1 ~226
α-tubulin ~50
i.
RPE1 p53-/- FRT/TR
Aph
siCTRL
siMDC1
DAPI TOPBP1
0.1626
Number of TOPBP1 foci/ prometaphase nuclei
0 10 20 30 40 50
siCTRL siMDC1
j.
RPE1 p53-/- FRT/TR
siCTRL siMDC1
MW (kDa)
MDC1 ~226
α-tubulin ~50

Supplementary Figure 2:
(a) Representative images and dot plot of TOPBP1 foci in DLD1 WT and DLD1 *BRCA2*$^{-/-}$ prometaphase cells synchronised with 60 ng/ml nocodazole for 2 hours (WT: n=74, *BRCA2*$^{-/-}$: n=73 from three independent experiments). (b) Representative images and dot plot of number of MDC1 foci in RPE1 *p53*$^{-/-}$ *PAC*$^{-/-}$ WT or *BRCA1*$^{-/-}$ cells synchronised with 60 ng/ml nocodazole for 2 hours (WT: n=86, *BRCA1*$^{-/-}$: n=109, from three independent experiments). (c). Representative images and dot plot of number of MDC1 foci in DLD1 WT and *BRCA2*$^{-/-}$ cells synchronised with 60 ng/ml nocodazole for 2 hours (WT: n=83, *BRCA2*$^{-/-}$: n=84 from three independent experiments). (d). Representative images and dot plot of number of MDC1 foci in RPE1 *p53*$^{-/-}$ FRT/TR cells untreated or treated with 400 nM aphidicolin for 18 hours followed by synchronisation with 60 ng/ml nocodazole for 2 hours (UT: n=79, Aph: n=80 from three independent experiments). (e) Representative images and dot plot of number of TOPBP1 foci in RPE1 *p53*$^{-/-}$ *PAC*$^{-/-}$ *BRCA1*$^{-/-}$ cells treated with siCTRL or siMDC1 followed by synchronisation with 60 ng/ml nocodazole for 2 hours (siCTRL: n=50, siMDC1: n=49 from two independent experiments). (f). Representative images and dot plot of number of TOPBP1 foci in DLD1 *BRCA2*$^{-/-}$ cells treated with siCTRL or siMDC1 followed by synchronisation with 60 ng/ml nocodazole for 2 hours (siCTRL: n=84, siMDC1: n=84 from three independent experiments). (g) Western blot analysis of DLD1 and DLD1 *BRCA2*$^{-/-}$ cells treated with siCTRL or siMDC1. (h) Western blot analysis of RPE1 *p53*$^{-/-}$ *PAC*$^{-/-}$ *BRCA1*$^{-/-}$ cells treated with siCTRL or siMDC1. (i). Representative images and dot plot of number of TOPBP1 foci in RPE1 *p53*$^{-/-}$ FRT/TR cells treated with siCTRL or siMDC1 followed by 400 nM aphidicolin for 18 hours followed by synchronisation with 60 ng/ml nocodazole for 2 hours (siCTRL: n=74, siMDC1: n=57 from three independent experiments). (j) Western blot analysis of RPE1 *p53*$^{-/-}$FRT/TR cells treated with siCTRL or siMDC1. Statistical significance for a, b, c, d, e, f and i was determined by two tailed unpaired t-test. Grey dots represent individual measurements; black dots indicate medians from individual experiments and bars represent the mean with SEM displayed; scale bars represent 10 μm. Source data are provided as a Source Data file.

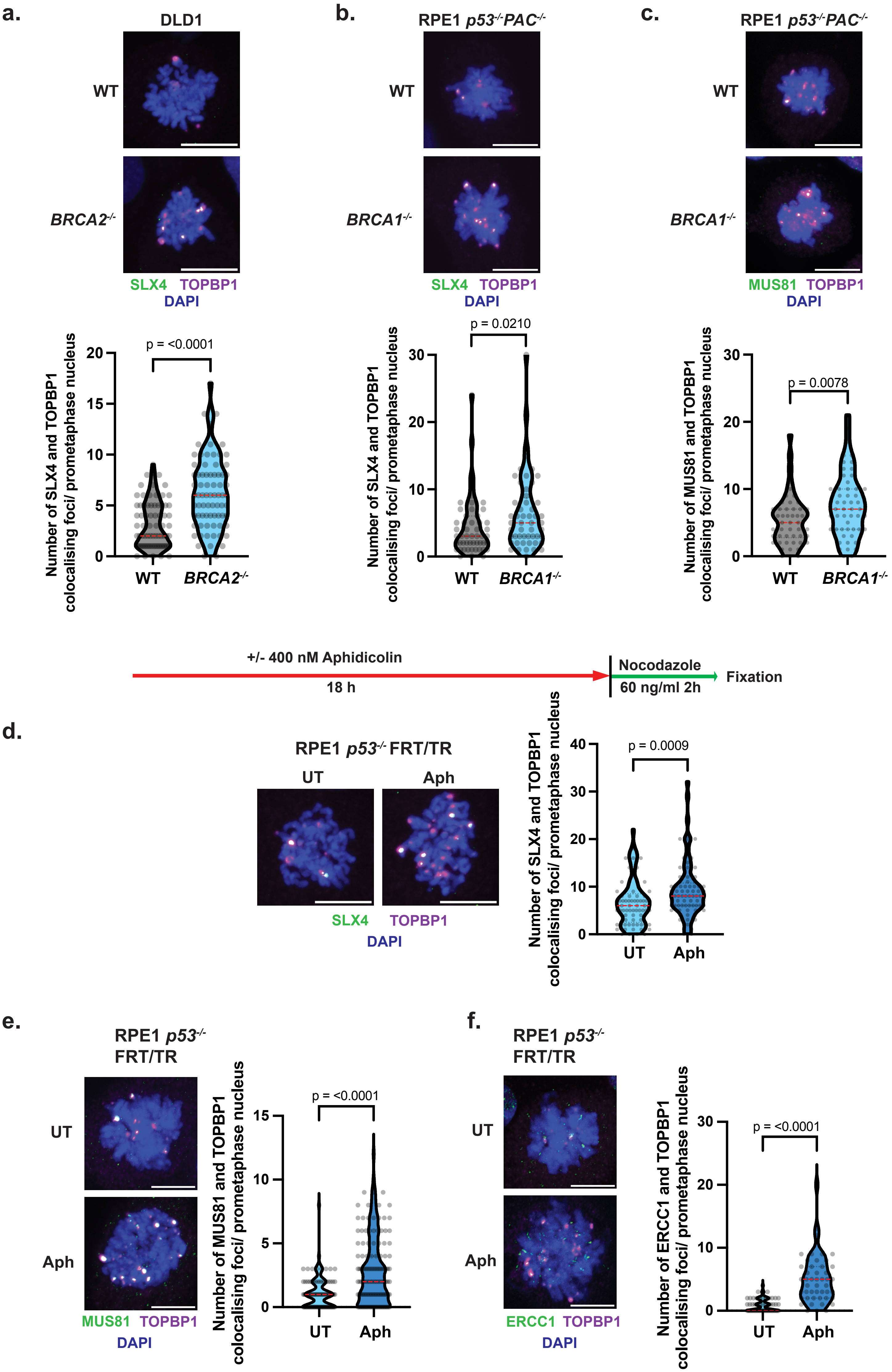
a.
DLD1
WT
BRCA2-/-
SLX4 TOPBP1
DAPI
Number of SLX4 and TOPBP1 colocalising foci/ prometaphase nucleus
p = <0.0001
WT
BRCA2-/-
b.
RPE1 p53-/-PAC-/-
WT
BRCA1-/-
SLX4 TOPBP1
DAPI
Number of SLX4 and TOPBP1 colocalising foci/ prometaphase nucleus
p = 0.0210
WT
BRCA1-/-
c.
RPE1 p53-/-PAC-/-
WT
BRCA1-/-
MUS81 TOPBP1
DAPI
Number of MUS81 and TOPBP1 colocalising foci/ prometaphase nucleus
p = 0.0078
WT
BRCA1-/-
+/- 400 nM Aphidicolin
18 h
Nocodazole
60 ng/ml 2h
Fixation
d.
RPE1 p53-/- FRT/TR
UT
Aph
SLX4 TOPBP1
DAPI
Number of SLX4 and TOPBP1 colocalising foci/ prometaphase nucleus
p = 0.0009
UT
Aph
e.
RPE1 p53-/- FRT/TR
UT
Aph
MUS81 TOPBP1
DAPI
Number of MUS81 and TOPBP1 colocalising foci/ prometaphase nucleus
p = <0.0001
UT
Aph
f.
RPE1 p53-/- FRT/TR
UT
Aph
ERCC1 TOPBP1
DAPI
Number of ERCC1 and TOPBP1 colocalising foci/ prometaphase nucleus
p = <0.0001
UT
Aph

Supplementary Figure 3:
(a) Representative images and violin plot of SLX4 and TOPBP1 colocalising foci in DLD1 WT and DLD1 *BRCA2*$^{-/-}$ prometaphase cells synchronised with 60 ng/ml nocodazole for 2 hours (WT: n=74, *BRCA2*$^{-/-}$: n=75 from three independent experiments). (b) Representative images and violin plot of SLX4 and TOPBP1 colocalising foci in RPE1 *p53*$^{-/-}$ *PAC*$^{-/-}$ *BRCA1*$^{-/-}$ prometaphase cells synchronised with 60 ng/ml nocodazole for 2 hours (WT: n=52, *BRCA1*$^{-/-}$: n=52 from two independent experiments). (c) Representative images and violin plot of MUS81 and TOPBP1 colocalising foci in RPE1 *p53*$^{-/-}$ *PAC*$^{-/-}$ *BRCA1*$^{-/-}$ prometaphase cells synchronised with 60 ng/ml nocodazole for 2 hours (WT: n=50, *BRCA1*$^{-/-}$: n=54 from two independent experiments). (d) Representative images and violin plot of SLX4 and TOPBP1 colocalising foci in RPE1 *p53*$^{-/-}$ FRT/TR prometaphase cells treated with or without aphidicolin then synchronised with 60 ng/ml nocodazole for 2 hours (UT: n=73, Aph: n=76 from three independent experiments). (e) Representative images and violin plot of MUS81 and TOPBP1 colocalising foci in RPE1 *p53*$^{-/-}$ FRT/TR prometaphase cells treated with or without 400 nM aphidicolin for 18 hours followed by 2 hour nocodazole treatment (UT: n= 96, Aph: n= 134, from two independent experiments). (f) Representative images and dot plot of ERCC1 and TOPBP1 colocalising foci in RPE1 *p53*$^{-/-}$ FRT/TR prometaphase cells treated with or without 400 nM aphidicolin for 18 hours followed by 2 hour nocodazole treatment (UT: n= 57, Aph: n= 47, from two independent experiments). Statistical significance in a, b, c, e and f was determined by Mann Whitney test, red line represents median, and scale bars represent 10 µm. Source data are provided as a Source Data file.

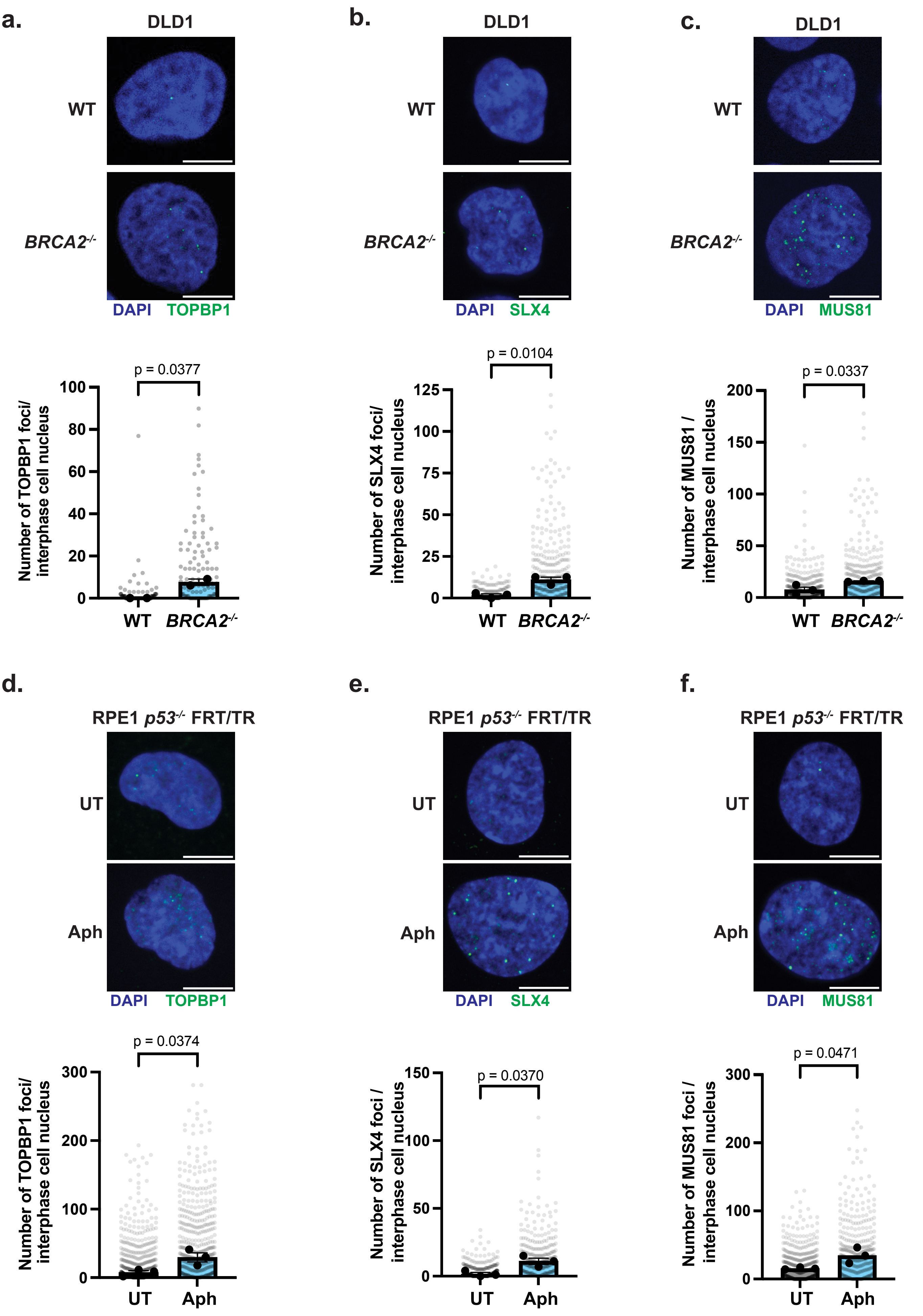
a.
DLD1
WT
BRCA2-/-
DAPI TOPBP1
p = 0.0377
Number of TOPBP1 foci/ interphase cell nucleus
100
80
60
40
20
0
WT BRCA2-/-
b.
DLD1
WT
BRCA2-/-
DAPI SLX4
p = 0.0104
Number of SLX4 foci/ interphase cell nucleus
125
100
75
50
25
0
WT BRCA2-/-
c.
DLD1
WT
BRCA2-/-
DAPI MUS81
p = 0.0337
Number of MUS81 / interphase cell nucleus
200
150
100
50
0
WT BRCA2-/-
d.
RPE1 p53-/- FRT/TR
UT
Aph
DAPI TOPBP1
p = 0.0374
Number of TOPBP1 foci/ interphase cell nucleus
300
200
100
0
UT Aph
e.
RPE1 p53-/- FRT/TR
UT
Aph
DAPI SLX4
p = 0.0370
Number of SLX4 foci / interphase cell nucleus
150
100
50
0
UT Aph
f.
RPE1 p53-/- FRT/TR
UT
Aph
DAPI MUS81
p = 0.0471
Number of MUS81 foci / interphase cell nucleus
300
200
100
0
UT Aph

Supplementary Figure 4:
(a) Representative images and dot plot of the number of TOPBP1 foci per interphase cell nucleus of DLD1 WT and *BRCA2*$^{-/-}$ cells (WT: n=200, *BRCA2*$^{-/-}$: n=200 from two individual experiments).(b) Representative images and dot plot of the number of SLX4 foci per interphase cell nucleus of DLD1 WT and *BRCA2*$^{-/-}$ cells (WT: n=300, *BRCA2*$^{-/-}$: n=300 from three individual experiments). (c) Representative images and dot plot of the number of MUS81 foci per interphase cell nucleus of DLD1 WT and *BRCA2*$^{-/-}$ cells (WT: n=299, *BRCA2*$^{-/-}$: n=306 from three individual experiments). (d) Representative images and dot plot of the number of TOPBP1 foci per interphase cell nucleus of RPE1 *p53*$^{-/-}$ FRT/TR cells treated without or with 400 nM aphidicolin for 18 hours (UT: n=592, Aph: n=459 from three individual experiments). (e) Representative images and dot plot of the number of SLX4 foci per interphase cell nucleus of RPE1 *p53*$^{-/-}$ FRT/TR cells treated without or with 400 nM aphidicolin for 18 hours (UT: n=338, Aph: n=302 from three individual experiments). (f) Representative images and dot plot of the number of MUS81 foci per interphase cell nucleus of RPE1 *p53*$^{-/-}$ FRT/TR cells treated without or with 400 nM aphidicolin for 18 hours (UT: n=443, Aph: n=360 from three individual experiments). a-f scale bars represent 10 μm, grey dots represent individual measurements, black dots indicate medians from individual experiments and bars represent the mean with SEM displayed, statistical significance was determined by two-tailed unpaired t-test. Source data are provided as a Source Data file.

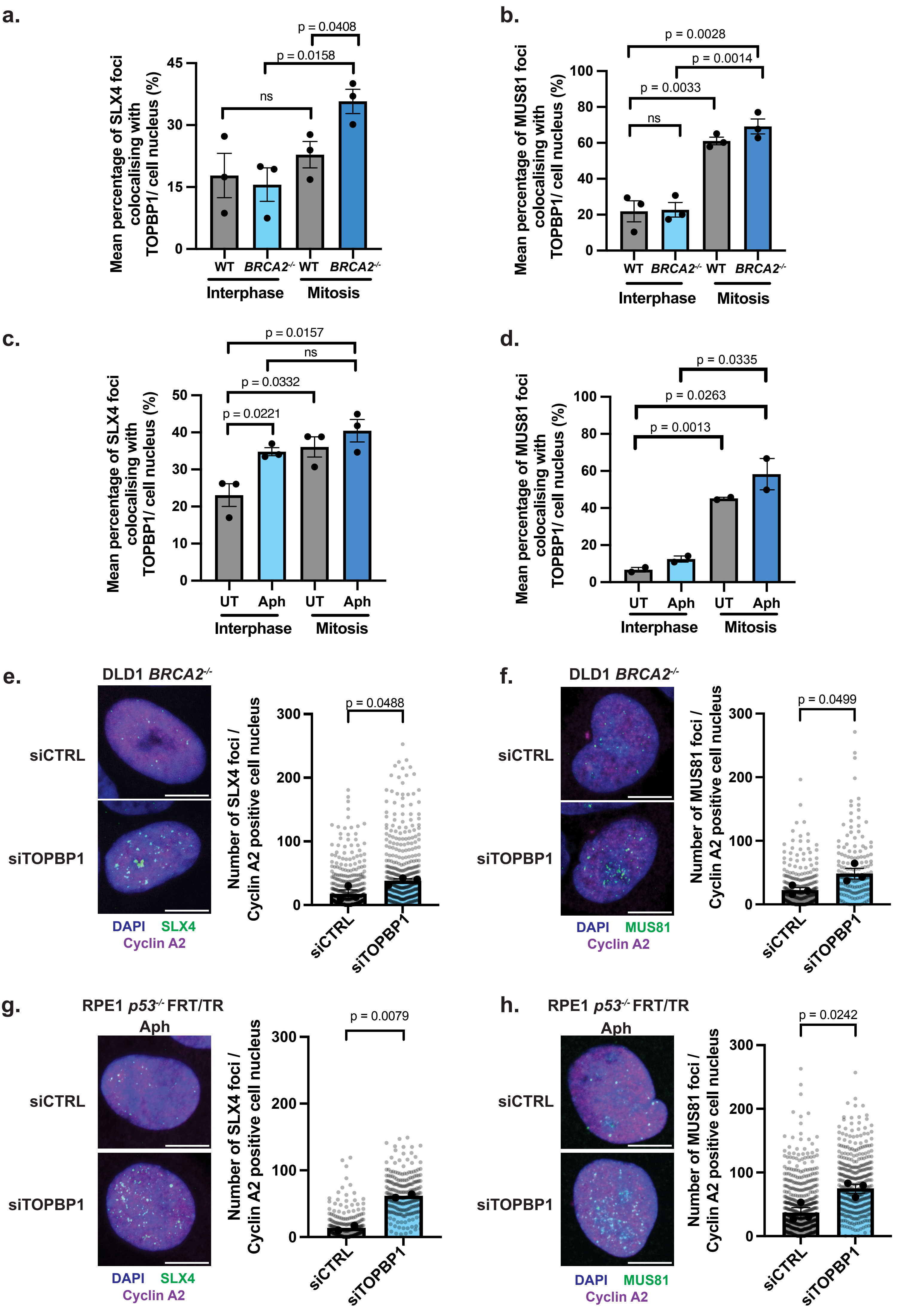
a.
Mean percentage of SLX4 foci colocalising with TOPBP1/ cell nucleus (%)
p = 0.0408
p = 0.0158
ns
WT
BRCA2-/-
WT
BRCA2-/-
Interphase
Mitosis
b.
Mean percentage of MUS81 foci colocalising with TOPBP1/ cell nucleus (%)
p = 0.0028
p = 0.0014
p = 0.0033
ns
WT
BRCA2-/-
WT
BRCA2-/-
Interphase
Mitosis
c.
Mean percentage of SLX4 foci colocalising with TOPBP1/ cell nucleus (%)
p = 0.0157
ns
p = 0.0332
p = 0.0221
UT
Aph
UT
Aph
Interphase
Mitosis
d.
Mean percentage of MUS81 foci colocalising with TOPBP1/ cell nucleus (%)
p = 0.0335
p = 0.0263
p = 0.0013
UT
Aph
UT
Aph
Interphase
Mitosis
e.
DLD1 BRCA2-/-
siCTRL
siTOPBP1
DAPI SLX4
Cyclin A2
Number of SLX4 foci / Cyclin A2 positive cell nucleus
p = 0.0488
siCTRL
siTOPBP1
f.
DLD1 BRCA2-/-
siCTRL
siTOPBP1
DAPI MUS81
Cyclin A2
Number of MUS81 foci / Cyclin A2 positive cell nucleus
p = 0.0499
siCTRL
siTOPBP1
g.
RPE1 p53-/- FRT/TR
Aph
siCTRL
siTOPBP1
DAPI SLX4
Cyclin A2
Number of SLX4 foci / Cyclin A2 positive cell nucleus
p = 0.0079
siCTRL
siTOPBP1
h.
RPE1 p53-/- FRT/TR
Aph
siCTRL
siTOPBP1
DAPI MUS81
Cyclin A2
Number of MUS81 foci / Cyclin A2 positive cell nucleus
p = 0.0242
siCTRL
siTOPBP1

Supplementary Figure 5

(a) Bar graph of the mean percentage of SLX4 foci colocalising with TOPBP1 in interphase compared to mitotic cell nuclei in DLD1 WT and *BRCA2*$^{-/-}$ cells (Interphase: WT: n=864, *BRCA2*$^{-/-}$: n=1046; Mitosis: WT: n=74, *BRCA2*$^{-/-}$: n=75 from three individual experiments). (b) Bar graph of the mean percentage of MUS81 foci colocalising with TOPBP1 in interphase compared to mitotic cell nuclei in DLD1 WT and *BRCA2*$^{-/-}$ cells (Interphase: WT: n=1057, *BRCA2*$^{-/-}$: n=1071; Mitosis: WT: n=74, *BRCA2*$^{-/-}$: n=73 from three individual experiments). (c) Bar graph of the mean percentage of SLX4 foci colocalising with TOPBP1 in interphase compared to mitotic cell nuclei in RPE1 *p53*$^{-/-}$ FRT/TR cells treated without or with 400 nM aphidicolin for 18 hours (Interphase: UT: n=553, Aph: n=367; Mitosis: UT: n=73, Aph: n=76 from three individual experiment). (d) Bar graph of the mean percentage of MUS81 foci colocalising with TOPBP1 in interphase compared to mitotic cell nuclei in RPE1 *p53*$^{-/-}$ FRT/TR cells treated without or with 400 nM aphidicolin for 18 hours (Interphase: UT: n=608, Aph: n=376; Mitosis: UT: n=96, Aph: n=133 from two individual experiments). a-d Black dots represent median percentage of colocalising foci per cell nucleus in each individual experiment; bars represent mean of these individual experiments with error bars depicting SEM. Statistical significance was determined by two-tailed unpaired t-test. (e) Representative images and dot plot of the number of SLX4 foci per Cyclin A2 positive interphase cell nucleus of DLD1 *BRCA2*$^{-/-}$ cells treated with siCTRL or siTOPBP1 (WT: n=384, *BRCA2*$^{-/-}$: n=357 from three individual experiments). (f) Representative images and dot plot of the number of MUS81 foci per Cyclin A2 positive interphase cell nucleus of DLD1 *BRCA2*$^{-/-}$ cells treated with siCTRL or siTOPBP1 (WT: n=239, *BRCA2*$^{-/-}$: n=161 from three individual experiments). (g) Representative images and dot plot of the number of SLX4 foci per Cyclin A2 positive interphase cell nucleus of RPE1 *p53*$^{-/-}$ FRT/TR cells treated with siCTRL or siTOPBP1 followed by 400 nM aphidicolin for 18 hours (UT: n=188, Aph: n=231 from two individual experiments). (h) Representative images and dot plot of the number of MUS81 foci per Cyclin A2 positive interphase cell nucleus of RPE1 *p53*$^{-/-}$ FRT/TR cells treated with siCTRL or siTOPBP1 followed by 400 nM aphidicolin for 18 hours (UT: n=513, Aph: n=368 from three individual experiments). e-h grey dots represent individual measurements, black dots indicate medians from individual experiments and bars represent the mean with SEM displayed, statistical significance was determined by two-tailed unpaired t-test, representative image scale bars represent 10 µm. Source data are provided as a Source Data file.

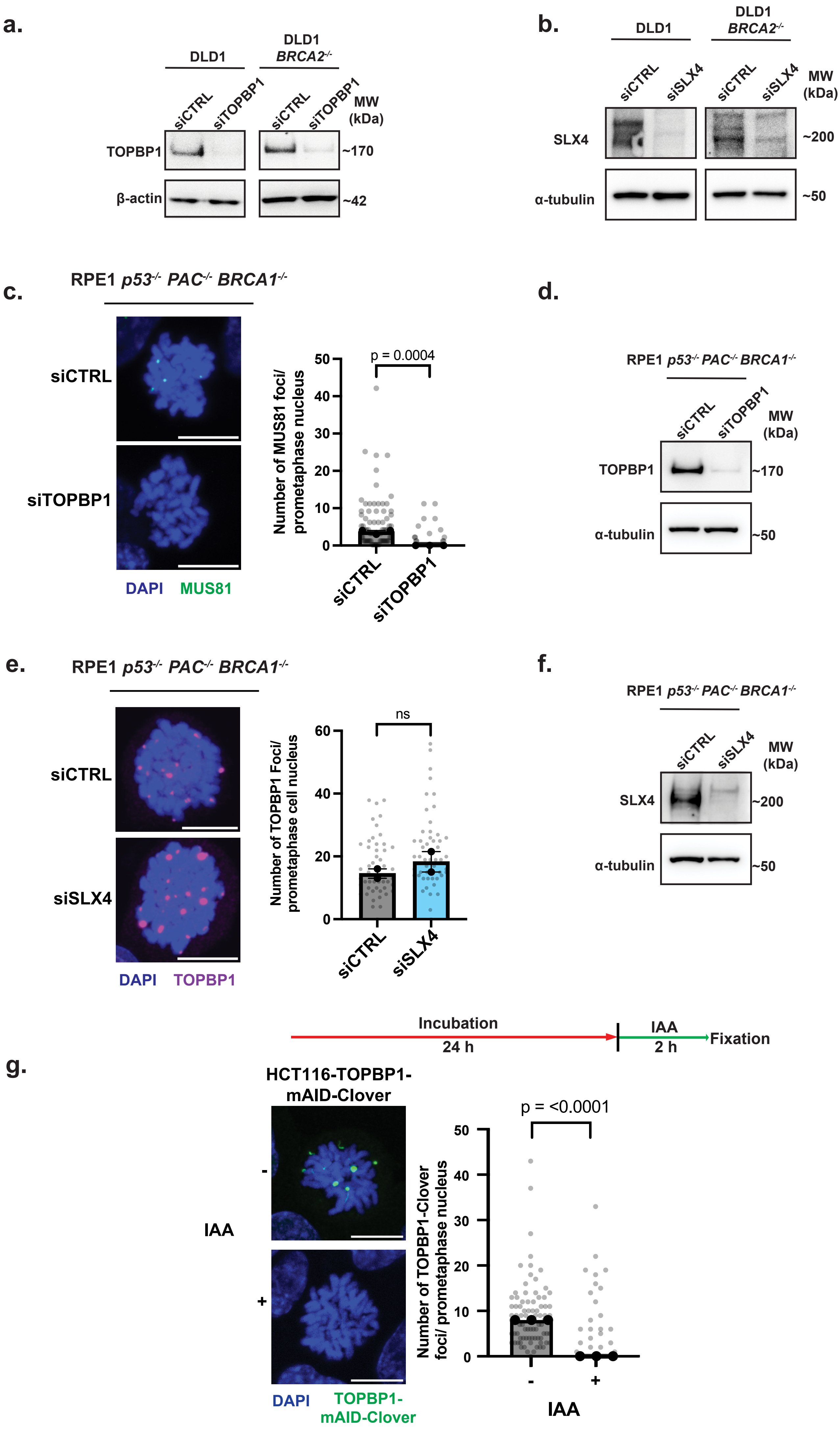
a.
DLD1
DLD1 BRCA2-/-
siCTRL
siTOPBP1
siCTRL
siTOPBP1
MW (kDa)
TOPBP1
~170
β-actin
~42
b.
DLD1
DLD1 BRCA2-/-
siCTRL
siSLX4
siCTRL
siSLX4
MW (kDa)
SLX4
~200
α-tubulin
~50
c.
RPE1 p53-/- PAC-/- BRCA1-/-
siCTRL
siTOPBP1
DAPI
MUS81
p = 0.0004
Number of MUS81 foci/ prometaphase nucleus
0
10
20
30
40
50
siCTRL
siTOPBP1
d.
RPE1 p53-/- PAC-/- BRCA1-/-
siCTRL
siTOPBP1
MW (kDa)
TOPBP1
~170
α-tubulin
~50
e.
RPE1 p53-/- PAC-/- BRCA1-/-
siCTRL
siSLX4
DAPI
TOPBP1
ns
Number of TOPBP1 Foci/ prometaphase cell nucleus
0
20
40
60
siCTRL
siSLX4
f.
RPE1 p53-/- PAC-/- BRCA1-/-
siCTRL
siSLX4
MW (kDa)
SLX4
~200
α-tubulin
~50
Incubation
24 h
IAA
2 h
Fixation
g.
HCT116-TOPBP1-mAID-Clover
IAA
-
+
DAPI
TOPBP1-mAID-Clover
p = <0.0001
Number of TOPBP1-Clover foci/ prometaphase nucleus
0
10
20
30
40
50
-
+
IAA

Supplementary Figure 6

(a) Western blot analysis of TOPBP1 in DLD1 WT or *BRCA2*$^{-/-}$ cells treated with siCTRL or siTOPBP1. (b) Western blot analysis of SLX4 in DLD1 WT or *BRCA2*$^{-/-}$ cells treated with siCTRL or siSLX4. (c) Representative images and dot plot of the number of MUS81 foci per prometaphase cell nucleus of RPE1 *p53*$^{-/-}$ *PAC*$^{-/-}$ *BRCA1*$^{-/-}$ cells treated with siCTRL or siTOPBP1 followed by synchronisation with 60 ng/ml nocodazole for two hours (siCTRL: n=72, siTOPBP1: n=50 from three individual experiments). (d) Western blot analysis of TOPBP1 in RPE1 *p53*$^{-/-}$ *PAC*$^{-/-}$ *BRCA1*$^{-/-}$ cells treated with siCTRL or siTOPBP1. (e) Representative images and dot plot of the number of TOPBP1 foci per prometaphase cell nucleus of RPE1 *p53*$^{-/-}$ *PAC*$^{-/-}$ *BRCA1*$^{-/-}$ cells treated with siCTRL or siSLX4 by synchronisation with 60 ng/ml nocodazole for two hours (siCTRL: n=50, siTOPBP1: n=51 from two individual experiments). (f) Western blot analysis of SLX4 in RPE1 *p53*$^{-/-}$ *PAC*$^{-/-}$ *BRCA1*$^{-/-}$ cells treated with siCTRL or siSLX4. (g). Representative images and dot plot of TOPBP1-mAID-Clover foci in HCT116-TOPBP1-mAID-Clover cells treated with or without IAA for 2 h. (-: n=86, +: n=88 from three independent experiments). In c, e and g grey dots represent individual measurements, black dots indicate medians from individual experiments and bars represent the mean with SEM displayed, statistical significance was determined by two-tailed unpaired t-test. Scale bars in c, e and g are equivalent to 10 µm. Source data are provided as a Source Data file.

a.

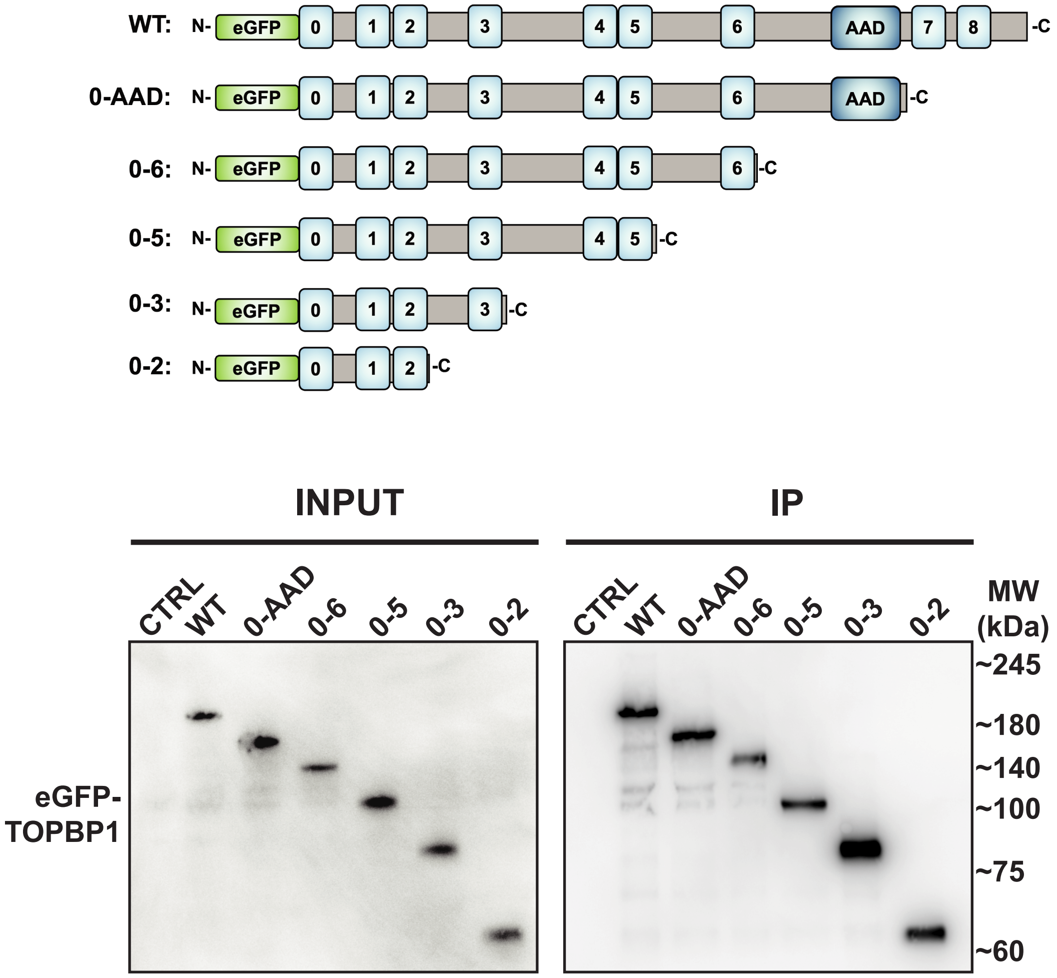
WT: N- eGFP 0 1 2 3 4 5 6 AAD 7 8 -C
0-AAD: N- eGFP 0 1 2 3 4 5 6 AAD -C
0-6: N- eGFP 0 1 2 3 4 5 6 -C
0-5: N- eGFP 0 1 2 3 4 5 -C
0-3: N- eGFP 0 1 2 3 -C
0-2: N- eGFP 0 1 2 -C
INPUT
IP
CTRL
WT
0-AAD
0-6
0-5
0-3
0-2
MW (kDa)
~245
~180
~140
~100
~75
~60
eGFP-TOPBP1

b.

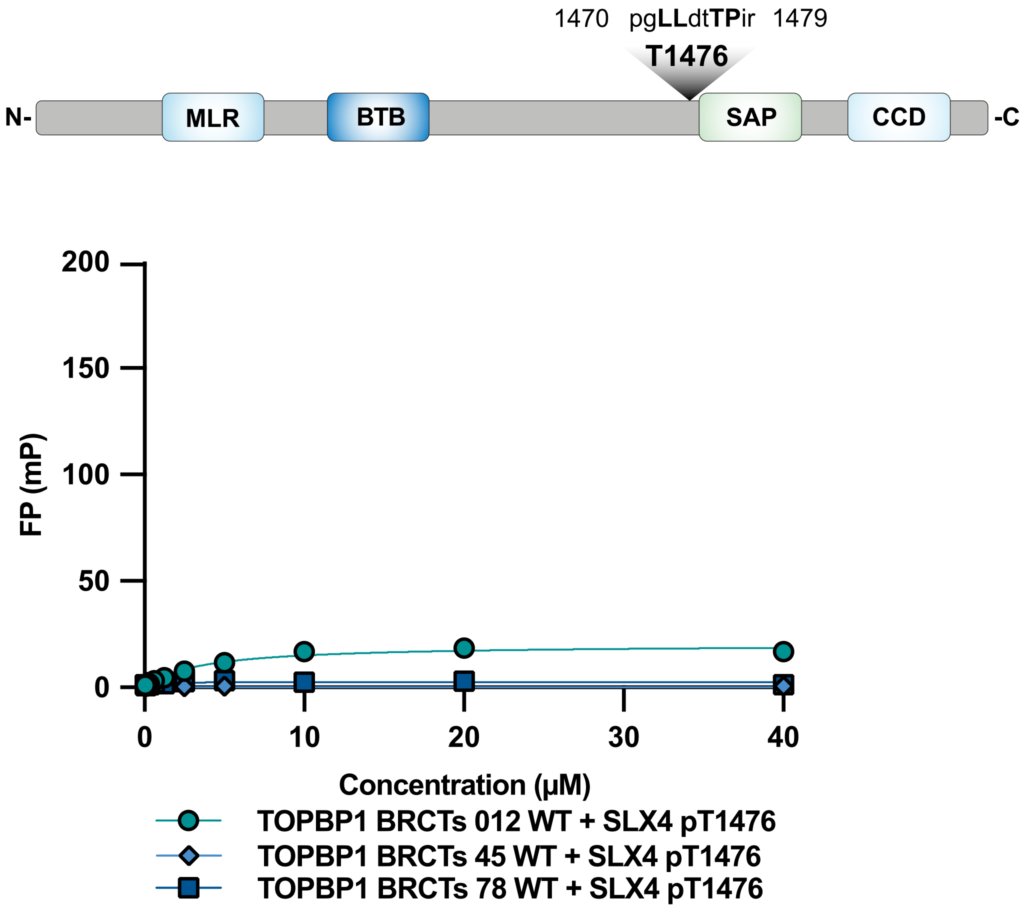
1470 pgLLdtTPir 1479
T1476
N-
MLR
BTB
SAP
CCD
-C
200
150
100
50
0
FP (mP)
0
10
20
30
40
Concentration (µM)
TOPBP1 BRCTs 012 WT + SLX4 pT1476
TOPBP1 BRCTs 45 WT + SLX4 pT1476
TOPBP1 BRCTs 78 WT + SLX4 pT1476

Supplementary Figure 7

(a) Domain organisation of eGFP-TOPBP1proteins analysed in and used in Figure. 3a. (b) SLX4 domain organisation and location of candidate TOPBP1 BRCT 1 interacting SLX4 pT1476 residue and fluorescence polarisation analysis of fluorescein labelled SLX4 pT1476 containing peptide in the presence of recombinant tandem BRCT containing TOPBP1 fragments (from three independent experiments). For all FP experiments raw data was fit with a specific and non-specific component, with the non-specific component subtracted before plotting. Source data are provided as a Source Data file.

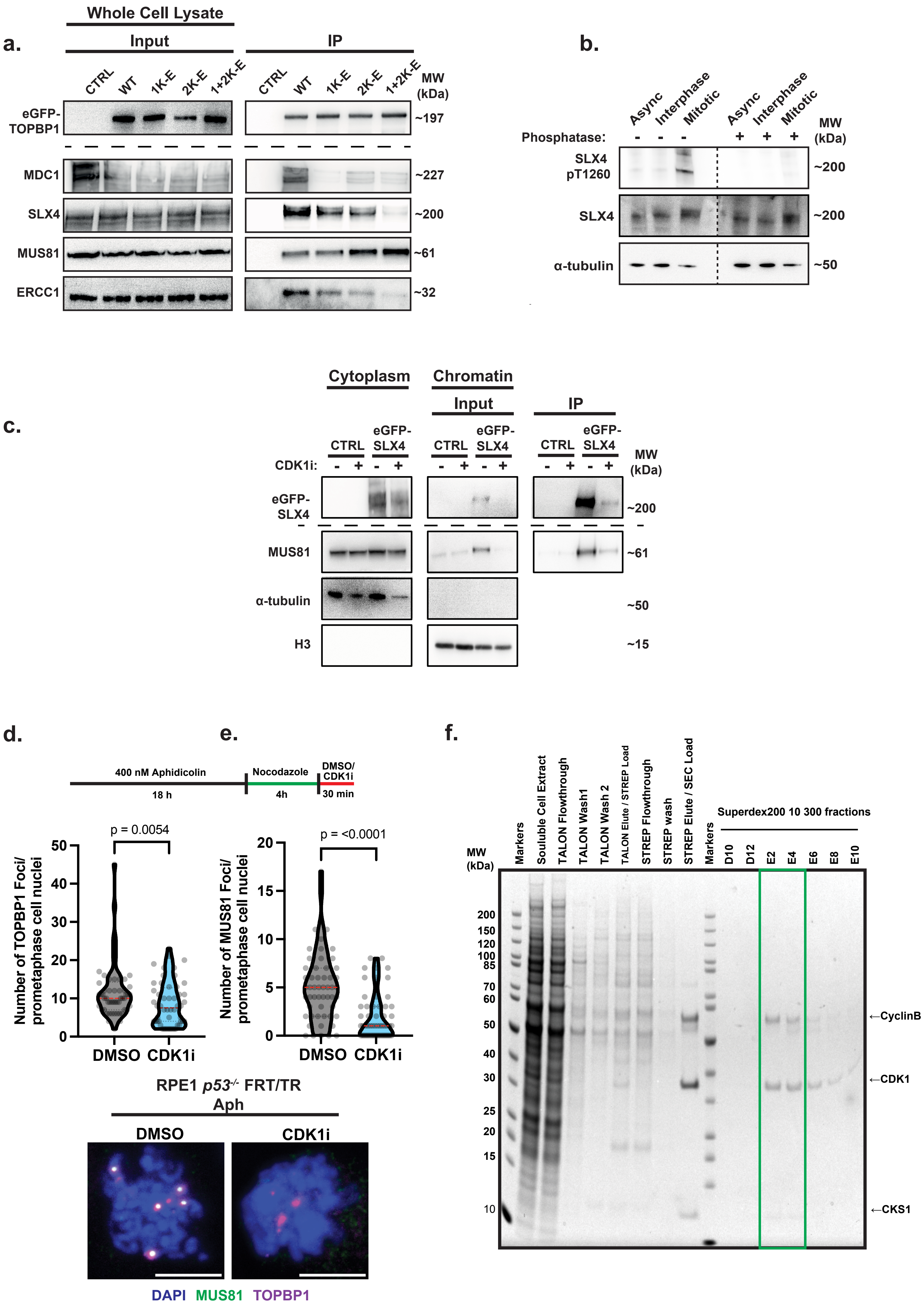
a.
Whole Cell Lysate
Input
IP
CTRL
WT
1K-E
2K-E
1+2K-E
MW (kDa)
eGFP-TOPBP1
~197
MDC1
~227
SLX4
~200
MUS81
~61
ERCC1
~32
b.
Async
Interphase
Mitotic
Phosphatase:
- - - + + +
SLX4 pT1260
~200
SLX4
~200
α-tubulin
~50
MW (kDa)
c.
Cytoplasm
Chromatin
Input
IP
CTRL
eGFP-SLX4
CDK1i:
- + - +
eGFP-SLX4
~200
MUS81
~61
α-tubulin
~50
H3
~15
MW (kDa)
d.
e.
400 nM Aphidicolin
18 h
Nocodazole
4h
DMSO/ CDK1i
30 min
p = 0.0054
Number of TOPBP1 Foci/ prometaphase cell nuclei
DMSO
CDK1i
p = <0.0001
Number of MUS81 Foci/ prometaphase cell nuclei
RPE1 p53-/- FRT/TR
Aph
DMSO
CDK1i
DAPI MUS81 TOPBP1
f.
Markers
Souluble Cell Extract
TALON Flowthrough
TALON Wash1
TALON Wash 2
TALON Elute / STREP Load
STREP Flowthrough
STREP wash
STREP Elute / SEC Load
Markers
Superdex200 10 300 fractions
D10
D12
E2
E4
E6
E8
E10
MW (kDa)
200
150
120
100
85
70
60
50
40
30
25
20
15
10
←CyclinB
←CDK1
←CKS1

Supplementary Figure 8

(a) Western blot analysis of MDC1, SLX4, MUS81 and ERCC1 in eGFP-TOPBP1 Co-IPs from HEK293TN cells treated with 100 ng/ml of nocodazole for 18h after transient transfection of eGFP-TOPBP1 WT, BRCT 1 (K155E), 2 (K250E) or 1+2 (K155E +K250E) expression constructs. Mock Co-IP from non-transfected HEK293TN cells was conducted as negative control (CTRL). (b) Western blot analysis of pT1260 SLX4 and total SLX4 in asynchronous, interphasic or cells isolated by mitotic shake off treated with or without λ phosphatase and shrimp intestinal phosphatase (dashed line indicates vertical separation to facilitate independent incubation of membrane with phosphatases (see methods)). (c) Western blot analysis of fractionated HEK293TN cells transfected with eGFP-SLX4, followed by synchronisation with 100 ng/ml nocodazole for 18 hours, then with DMSO or 7 µM RO-3306 (CDK1i) for 30 min. d-e Representative images and violin plot of number of TOPBP1 (d) and MUS81 (e) foci per prometaphase nucleus of RPE1 *p53*$^{-/-}$ FRT/TR cells treated with 400 nM aphidicolin for 18 hours followed by 60 ng/ ml nocodazole for 4 hours, then DMSO or 5 µM RO-3306 (CDK1i) for 30 min ((d) DMSO: n=54, CDK1i: n=52; (e) DMSO: n=54, CDK1i: n=60 from two individual experiments, statistical significance was determined by Mann-Whitney test). Grey dots represent individual values, red line indicates median, and scale bars represent 10 µm. (f) SDS-PAGE showing purified recombinant CDK1-Cyclin B-CKS1 complex fractions E2-E4 were pooled for use in assays. Source data are provided as a Source Data file.

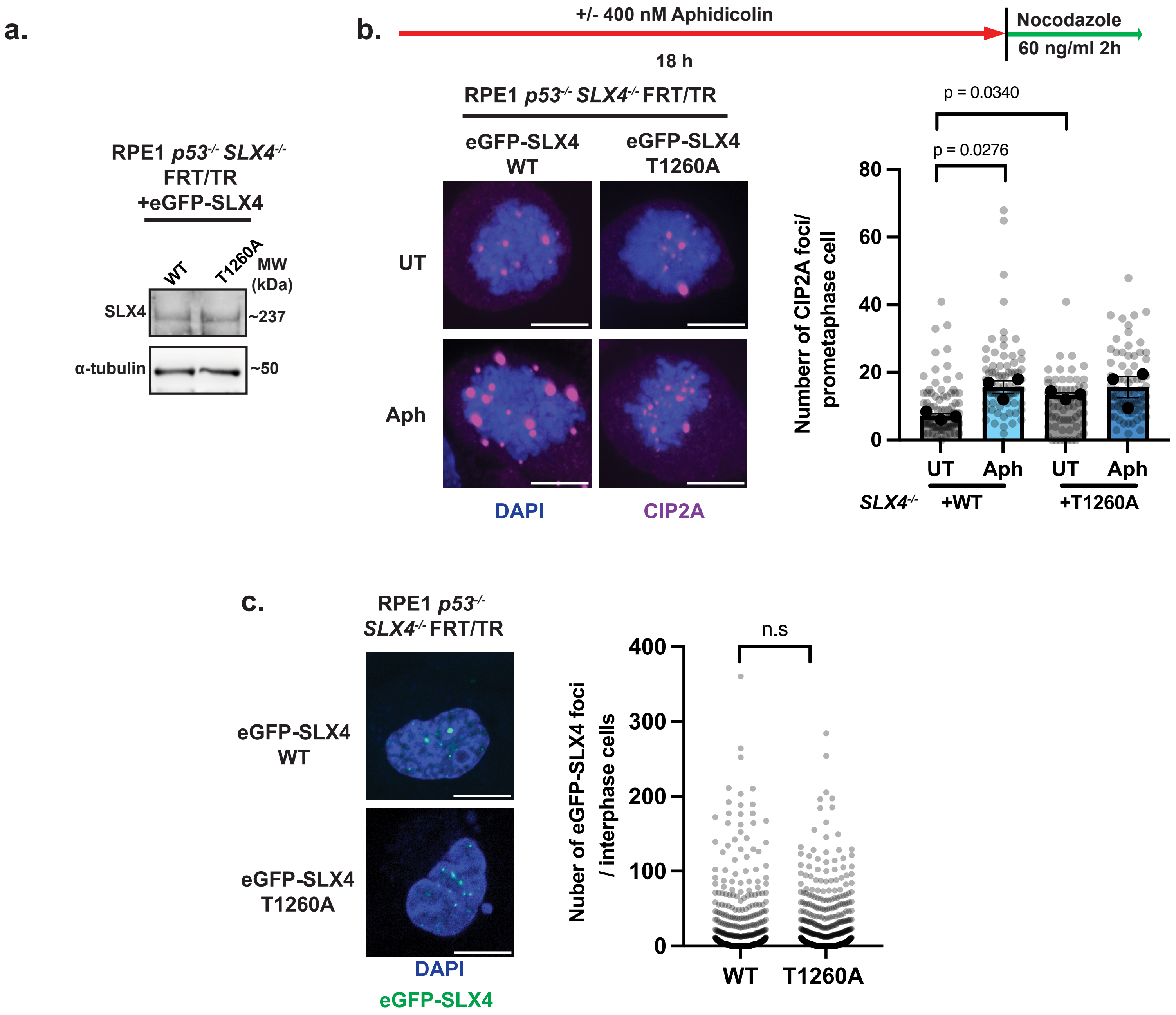
a.
RPE1 p53-/- SLX4-/- FRT/TR +eGFP-SLX4
WT
T1260A
MW (kDa)
SLX4
~237
α-tubulin
~50
b.
+/- 400 nM Aphidicolin
18 h
Nocodazole 60 ng/ml 2h
RPE1 p53-/- SLX4-/- FRT/TR
eGFP-SLX4 WT
eGFP-SLX4 T1260A
UT
Aph
DAPI
CIP2A
p = 0.0340
p = 0.0276
Numberr of CIP2A foci/ prometaphase cell
0
20
40
60
80
UT
Aph
UT
Aph
SLX4-/-
+WT
+T1260A
c.
RPE1 p53-/- SLX4-/- FRT/TR
eGFP-SLX4 WT
eGFP-SLX4 T1260A
DAPI
eGFP-SLX4
n.s
Nuber of eGFP-SLX4 foci / interphase cells
0
100
200
300
400
WT
T1260A

Supplementary Figure 9

(a) Western blot analysis of eGFP-SLX4 expression in RPE1 *p53*$^{-/-}$ *SLX4*$^{-/-}$ FRT/TR + eGFP-SLX4 WT and eGFP-SLX4 T1260A cells treated with 10 ng/ml doxycycline for 24 hours as treated in advance of all experiments using cell lines. (b) Representative images and dot plot of CIP2A foci in RPE1 *p53*$^{-/-}$ *SLX4*$^{-/-}$ FRT/TR +eGFP-SLX4 WT/T1260A prometaphase cells treated with 10 ng/ml doxycycline for 24 hours followed by treatment with or without 400 nM aphidicolin for 18 hours followed by 60 ng/ml nocodazole synchronisation (+eGFP-SLX4 WT (UT: n=85, Aph: n=76) +eGFP-SLX4 T1260A (UT: n=70, Aph: n=76) from three independent experiments, statistical significance was determined using two way ANOVA). Grey dots represent individual measurements; black dots represent medians from individual experiments and bars show the mean with SEM displayed. (c) Representative images and dot plot of eGFP-SLX4 or eGFP-SLX4 T1260A foci in interphasic RPE1 *p53*$^{-/-}$ *SLX4*$^{-/-}$ FRT/TR cells (+eGFP-SLX4 WT (UT: n=565, Aph: n=369) +eGFP-SLX4 T1260A (UT: n=639, Aph: n=421) from three independent experiments, statistical significance was determined by Mann-Whitney test. Grey dots show individual measurements. Scale bars in b and c represent 10 µm. Source data are provided as a Source Data file.

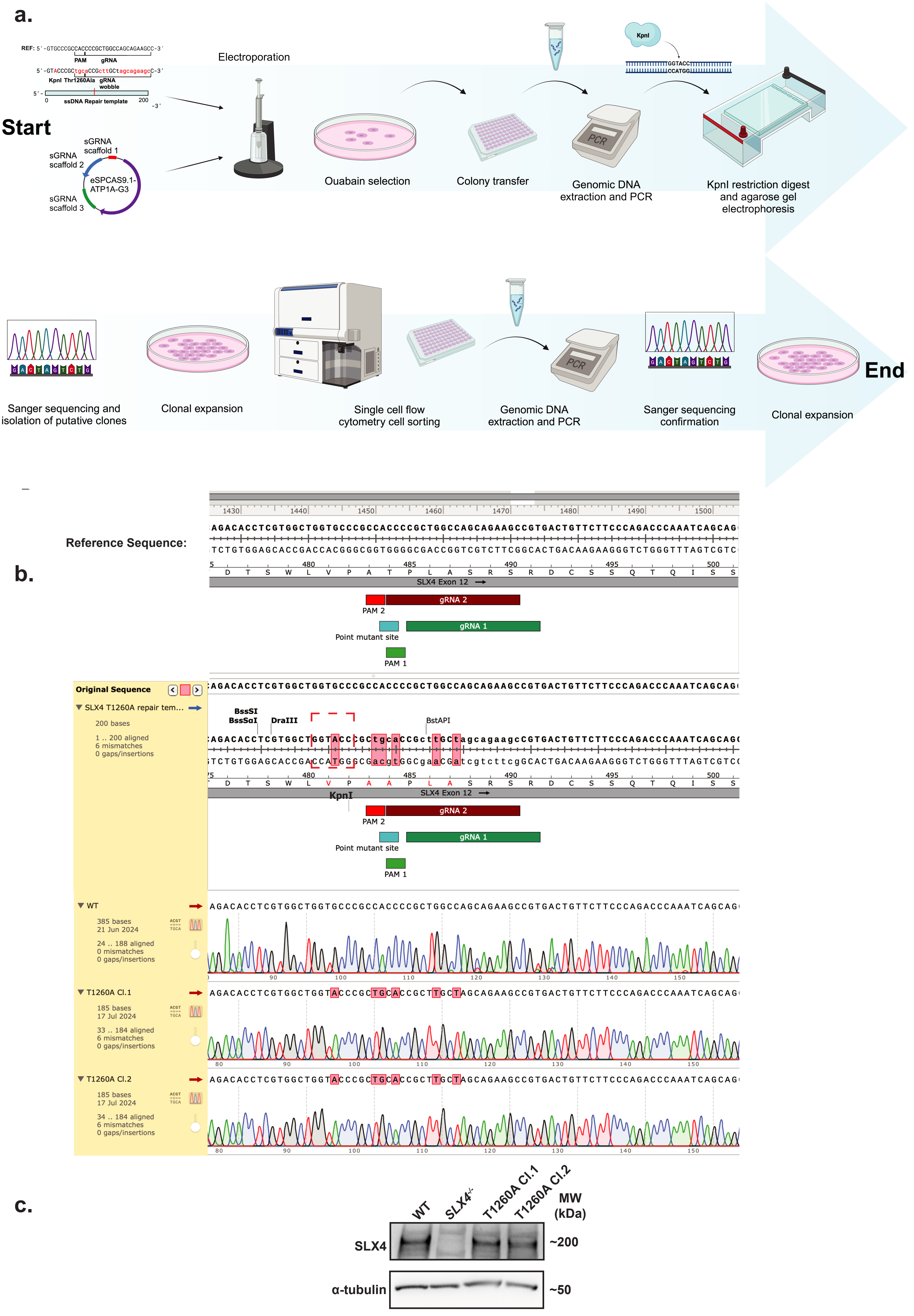

a.
Start
Electroporation
Ouabain selection
Colony transfer
Genomic DNA extraction and PCR
KpnI restriction digest and agarose gel electrophoresis
KpnI
ssDNA Repair template
sGRNA scaffold 1
sGRNA scaffold 2
sGRNA scaffold 3
eSPCAS9.1-ATP1A-G3
Sanger sequencing and isolation of putative clones
Clonal expansion
Single cell flow cytometry cell sorting
Genomic DNA extraction and PCR
Sanger sequencing confirmation
Clonal expansion
End
b.
Reference Sequence:
SLX4 Exon 12
gRNA 2
PAM 2
gRNA 1
Point mutant site
PAM 1
Original Sequence
SLX4 T1260A repair tem...
200 bases
1 .. 200 aligned
6 mismatches
0 gaps/insertions
BssSI
BssSαI
DraIII
BstAPI
KpnI
WT
385 bases
21 Jun 2024
24 .. 188 aligned
0 mismatches
0 gaps/insertions
T1260A Cl.1
185 bases
17 Jul 2024
33 .. 184 aligned
6 mismatches
0 gaps/insertions
T1260A Cl.2
185 bases
17 Jul 2024
34 .. 184 aligned
6 mismatches
0 gaps/insertions
c.
WT
SLX4-/-
T1260A Cl.1
T1260A Cl.2
MW (kDa)
SLX4
~200
α-tubulin
~50

Supplementary Figure 10

(a) Schematic of RPE1 *p53*$^{-/-}$ FRT/TR SLX4 Thr1260Ala knock-in cell line generation by ATP1A_editing ouabain coselection (Created in BioRender. Martin, P. (2025) https://BioRender.com/c82w495). (b). Multiple-sequence alignment of the SLX4 reference sequence with translated native amino acids and guide RNA target sites labelled, below is alignment of the SLX4 Thr1260Ala repair template used, containing the Thr1260Ala point mutation, silent mutation of guide RNA recognition sites and silent mutation upstream to create a KpnI restriction site (highlighted by red dashed box). Below shows sanger sequencing traces of WT, Thr1260Ala Cl.1 and Cl.2 PCR products amplified from genomic DNA using primers spanning the edited locus. (c) Western blot analysis of SLX4 expression in RPE1 *p53*$^{-/-}$ FRT/TR WT, *SLX4*$^{-/-}$, SLX4 T1260A knock-in clones (Cl.) 1 and 2. Source data are provided as a Source Data file.

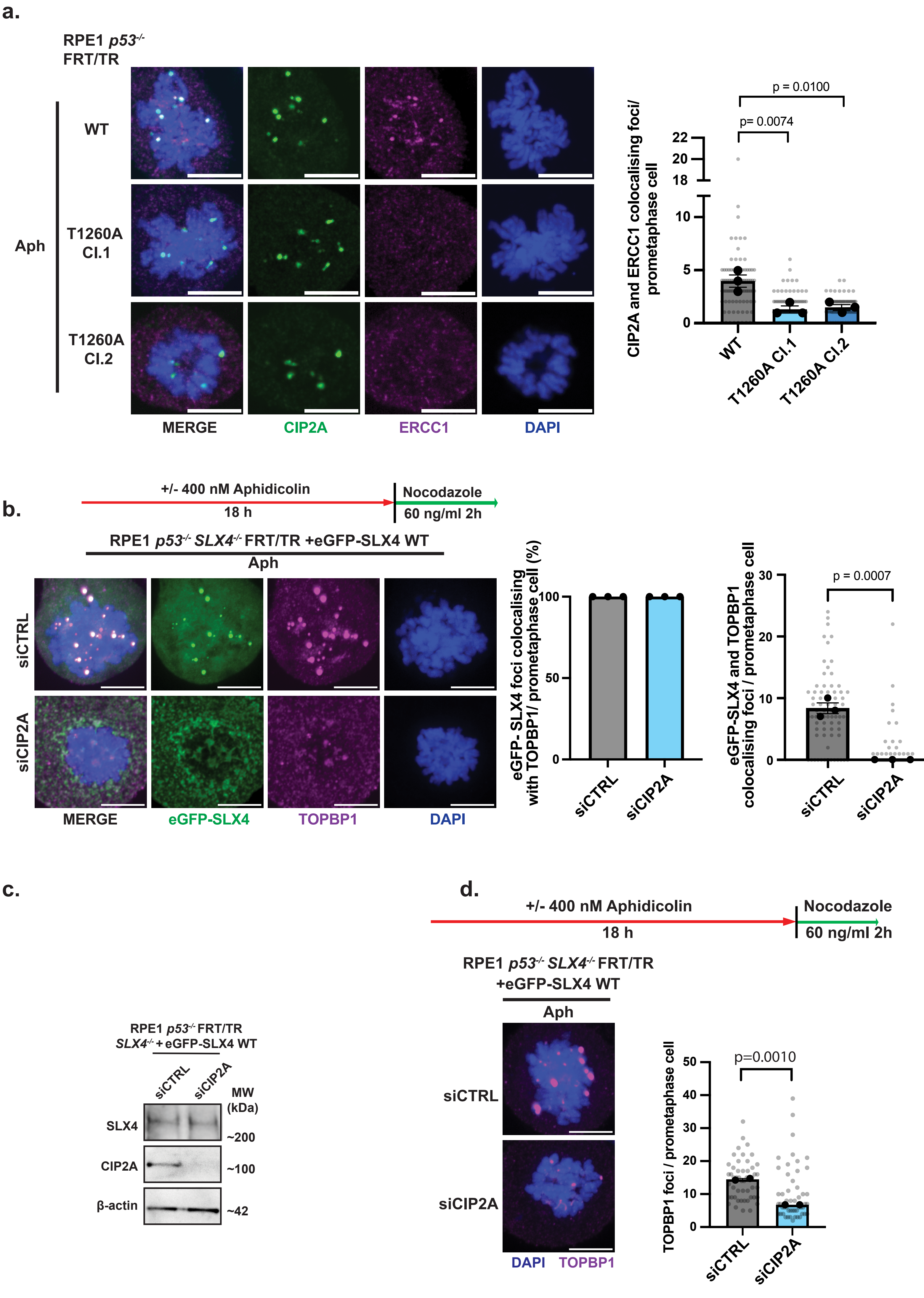
a.
RPE1 p53-/-
FRT/TR
WT
Aph
T1260A
Cl.1
T1260A
Cl.2
MERGE
CIP2A
ERCC1
DAPI
CIP2A and ERCC1 colocalising foci/
prometaphase cell
p = 0.0100
p= 0.0074
22
20
18
10
5
0
WT
T1260A Cl.1
T1260A Cl.2
b.
+/- 400 nM Aphidicolin
18 h
Nocodazole
60 ng/ml 2h
RPE1 p53-/- SLX4-/- FRT/TR +eGFP-SLX4 WT
Aph
siCTRL
siCIP2A
MERGE
eGFP-SLX4
TOPBP1
DAPI
eGFP- SLX4 foci colocalising
with TOPBP1/ prometaphase cell (%)
100
50
0
siCTRL
siCIP2A
eGFP-SLX4 and TOPBP1
colocalising foci / prometaphase cell
p = 0.0007
30
20
10
0
siCTRL
siCIP2A
c.
RPE1 p53-/- FRT/TR
SLX4-/- + eGFP-SLX4 WT
siCTRL
siCIP2A
MW
(kDa)
SLX4
~200
CIP2A
~100
β-actin
~42
d.
+/- 400 nM Aphidicolin
18 h
Nocodazole
60 ng/ml 2h
RPE1 p53-/- SLX4-/- FRT/TR
+eGFP-SLX4 WT
Aph
siCTRL
siCIP2A
DAPI
TOPBP1
TOPBP1 foci / prometaphase cell
p=0.0010
50
40
30
20
10
0
siCTRL
siCIP2A

Supplementary Figure 11

(a) Representative images and dot plot of ERCC1 and CIP2A colocalising foci in RPE1 *p53*$^{-/-}$ FRT/TR WT and SLX4 T1260A knock in prometaphase cells, treated with 400 nM aphidicolin for 18 hours followed by synchronisation with 60 ng/ml nocodazole for 2 hours (Parental WT: n=88, T1260A Cl.1: n= 80 T1260A Cl.2 n=76, from three independent experiments, statistical significance was determined by one way ANOVA). Grey dots represent individual measurements; black dots represent medians from individual experiments and bars show the mean with SEM displayed. (b) Representative images, bar plot showing the mean percentage of eGFP-SLX4 WT foci colocalising with CIP2A per RPE1 *p53*$^{-/-}$ *SLX4*$^{-/-}$ FRT/TR +eGFP-SLX4 WT/ T1260A per prometaphase cell after induction with 10 ng/ml doxycycline for 24 hours followed by treatment with or without 400 nM/ 18 h aphidicolin followed by synchronisation with 60 ng/ml nocodazole for 2 hours (UT: n=85, Aph: n=76), and a dot plot showing the number of eGFP-SLX4 WT or eGFP-SLX4 T1260A foci colocalising with CIP2A foci per prometaphase cell after treatment as above (eGFP-SLX4 WT (UT: n=85, Aph: n=76); and eGFP-SLX4 T1260A (UT: n=70, Aph: n=76) from three independent experiments, statistical significance was determined by two-way ANOVA). (c) Western blot analysis of SLX4 and CIP2A in RPE1 *p53*$^{-/-}$ *SLX4*$^{-/-}$ FRT/TR cells treated with 10 ng/ml doxycycline for 24 hours to induce eGFP-SLX4 WT expression, treated with siCTRL or siCIP2A. (d) Representative images of dot plot of TOPBP1 foci in RPE1 *p53*$^{-/-}$ *SLX4*$^{-/-}$ FRT/TR eGFP-SLX4 WT expressing prometaphase cells treated with siCTRL or siCIP2A, then 10 ng/ml doxycycline for 24 hours, followed by 400 nM aphidicolin for 18 hours then synchronised with 60 ng/ml nocodazole for 2 hours (siCTRL: n=74, siSLX4: n=73 from three independent experiments, statistical significance was determined using two tailed unpaired t-test). In a, b and d grey dots represent individual measurements; black dots represent the medians of each experiment and bars show the mean with error bars showing SEM and scale bar represents 10 µm. Source data are provided as a Source Data file.

a.

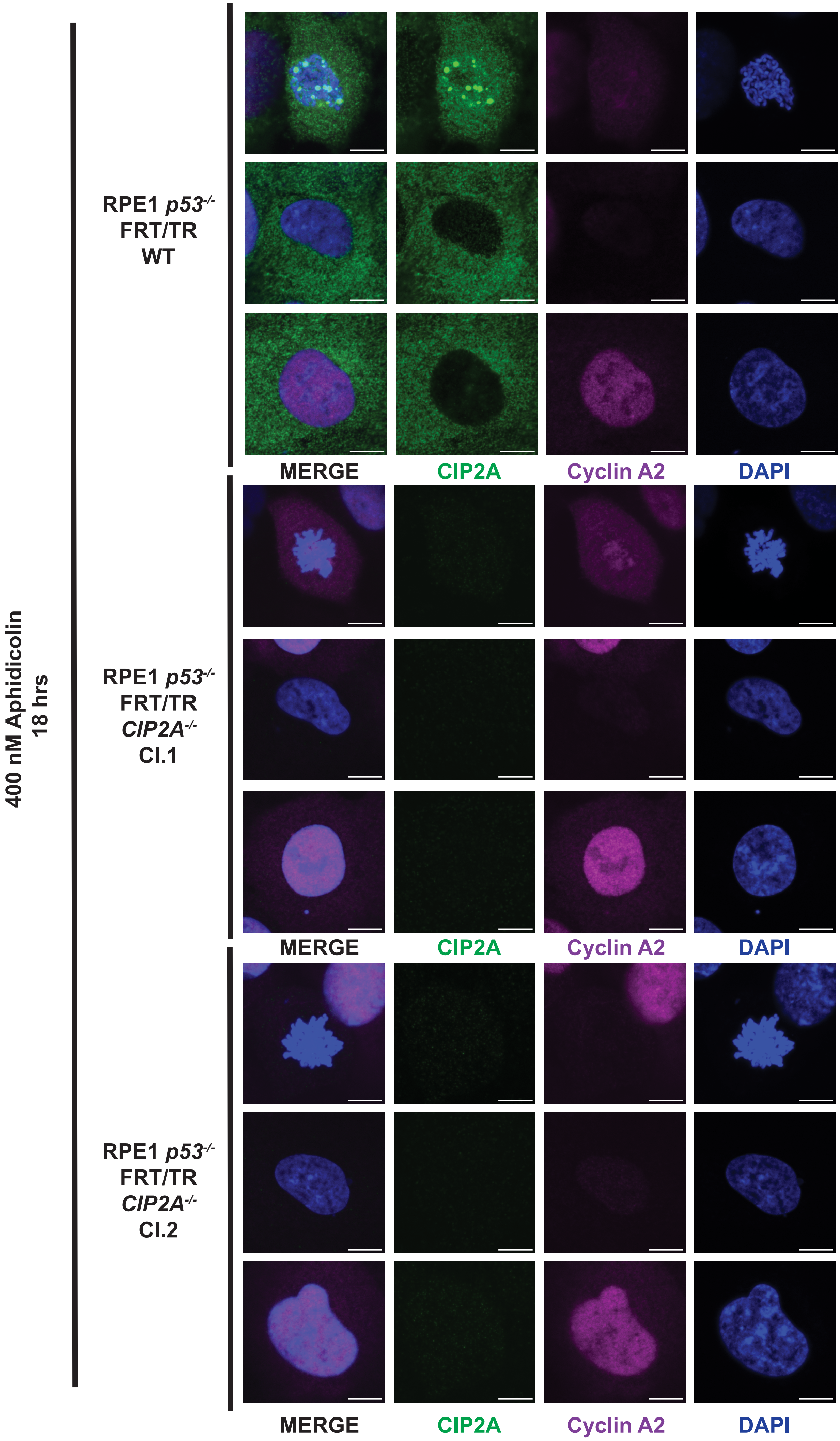
400 nM Aphidicolin
18 hrs
RPE1 p53-/-
FRT/TR
WT
MERGE
CIP2A
Cyclin A2
DAPI
RPE1 p53-/-
FRT/TR
CIP2A-/-
Cl.1
MERGE
CIP2A
Cyclin A2
DAPI
RPE1 p53-/-
FRT/TR
CIP2A-/-
Cl.2
MERGE
CIP2A
Cyclin A2
DAPI

Supplementary Figure 12

(a) Representative images of CIP2A localisation in prometaphase mitotic cells, Cyclin A2 negative and positive interphase RPE1 *p53*$^{-/-}$ FRT/TR WT cells and *CIP2A*$^{-/-}$ cells treated with 400 nM aphidicolin for 18 hours. Scale bars represent 10 μm.

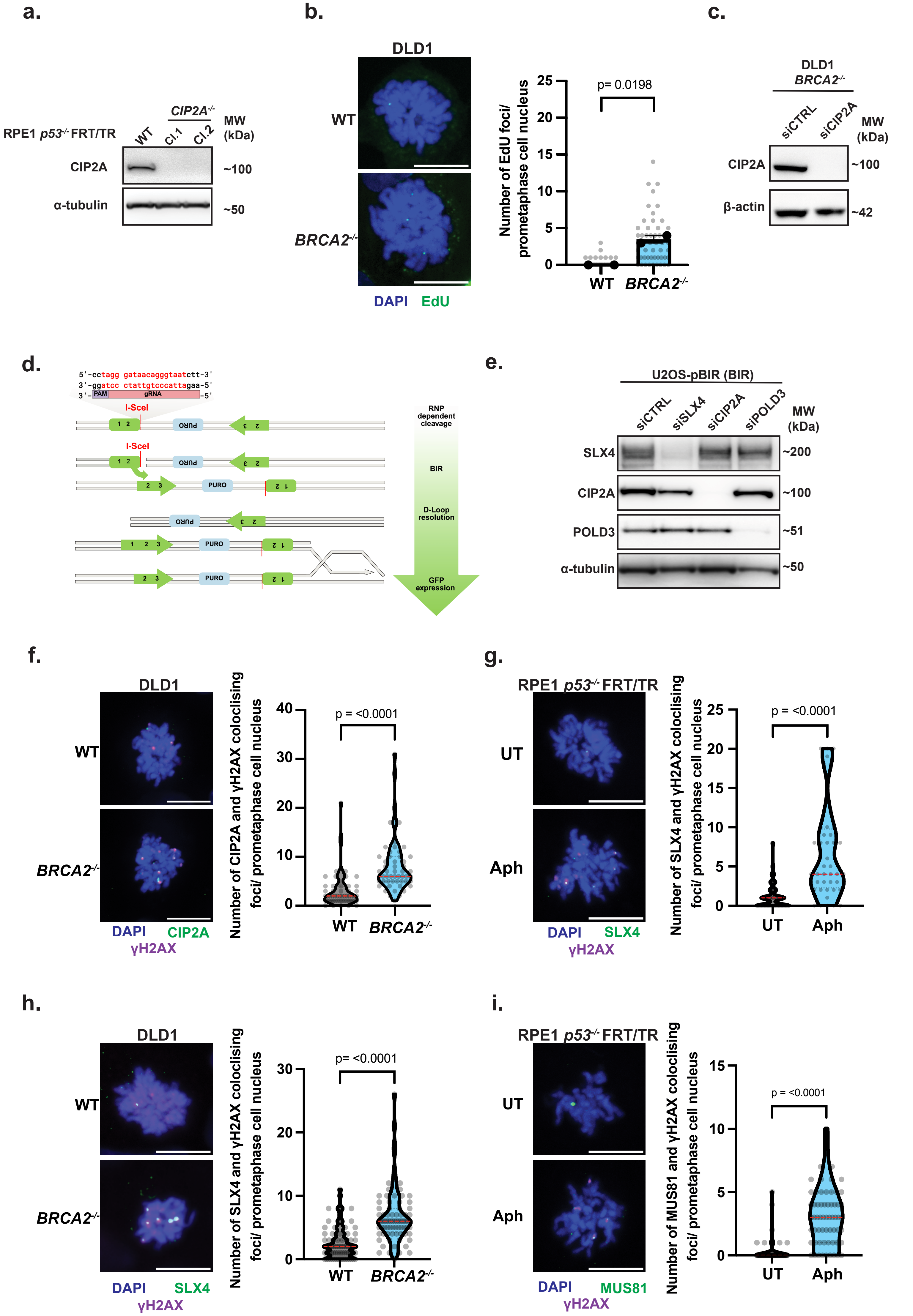

a.
RPE1 p53-/- FRT/TR
CIP2A-/-
WT
Cl.1
Cl.2
MW (kDa)
CIP2A
~100
α-tubulin
~50
b.
DLD1
WT
BRCA2-/-
DAPI
EdU
Number of EdU foci/ prometaphase cell nucleus
p= 0.0198
0
5
10
15
20
25
WT
BRCA2-/-
c.
DLD1 BRCA2-/-
siCTRL
siCIP2A
MW (kDa)
CIP2A
~100
β-actin
~42
d.
5'-cctagg gataacagggtaatctt-3'
3'-ggatcc ctattgtcccattagaa-5'
3'-PAM gRNA-5'
I-SceI
PURO
RNP dependent cleavage
BIR
D-Loop resolution
GFP expression
e.
U2OS-pBIR (BIR)
siCTRL
siSLX4
siCIP2A
siPOLD3
MW (kDa)
SLX4
~200
CIP2A
~100
POLD3
~51
α-tubulin
~50
f.
DLD1
WT
BRCA2-/-
DAPI
CIP2A
γH2AX
Number of CIP2A and γH2AX colocising foci/ prometaphase cell nucleus
p = <0.0001
0
10
20
30
40
WT
BRCA2-/-
g.
RPE1 p53-/- FRT/TR
UT
Aph
DAPI
SLX4
γH2AX
Number of SLX4 and γH2AX colocising foci/ prometaphase cell nucleus
p = <0.0001
0
5
10
15
20
25
UT
Aph
h.
DLD1
WT
BRCA2-/-
DAPI
SLX4
γH2AX
Number of SLX4 and γH2AX colocising foci/ prometaphase cell nucleus
p= <0.0001
0
10
20
30
WT
BRCA2-/-
i.
RPE1 p53-/- FRT/TR
UT
Aph
DAPI
MUS81
γH2AX
Number of MUS81 and γH2AX colocising foci/ prometaphase cell nucleus
p = <0.0001
0
5
10
15
UT
Aph

Supplementary Figure 13

(a) Western blot analysis of CIP2A in RPE1 *p53*$^{-/-}$ FRT/TR WT and *CIP2A*$^{-/-}$ cells. (b) Representative images and dot plot of EdU foci in prometaphase DLD1 WT and *BRCA2*$^{-/-}$ cells (WT: n=52, *BRCA2*$^{-/-}$: n= 52, from two independent experiments, statistical significance was determined by two tailed unpaired t-test). Grey dots represent individual measurements, black dots the median from each individual experiment and the bars represent the means with SEM displayed. Scale bars are equivalent to 10 µm. (c) Western blot analysis of DLD1 *BRCA2*$^{-/-}$ treated with siCTRL or siCIP2A. (d) Schematic of the pBIR reporter system (Created in BioRender. Martin, P. (2025) https://BioRender.com/o75g269). (e) Western blot analysis of U2OS pBIR reporter cells treated with siCTRL, siSLX4, siCIP2A or siPOLD3. (f) Representative images and violin plot of the number of CIP2A and γH2AX colocalising foci per prometaphase DLD1 WT or DLD1 *BRCA2*$^{-/-}$ cell nucleus synchronised with 60 ng/ml nocodazole for 2 hours (WT: n=55, *BRCA2*$^{-/-}$: n= 53 from two individual experiments). (g). Representative images and violin plot of the number of SLX4 and γH2AX colocalising foci per prometaphase RPE1 *p53*$^{-/-}$ FRT/TR cell nucleus, treated without or with 400 nM aphidicolin for 18 hours followed by 60 ng/ml nocodazole for 2 hours (UT: n=54, Aph: n= 44 from two individual experiments). (h) Representative images and violin plot of the number of SLX4 and γH2AX colocalising foci per prometaphase DLD1 WT or DLD1 *BRCA2*$^{-/-}$ cell nucleus synchronised with 60 ng/ml nocodazole for 2 hours (WT: n=76, *BRCA2*$^{-/-}$: n= 80 from three individual experiments). (i) Representative images and violin plot of the number of MUS81 and γH2AX colocalising foci per prometaphase RPE1 *p53*$^{-/-}$ FRT/TR cell nucleus, treated without or with 400 nM aphidicolin for 18 hours followed by 60 ng/ml nocodazole for 2 hours (UT: n=76, Aph: n= 63 from three individual experiments). In f-i statistical significance was determined by Mann-Whitney test, grey dots represent individual values, red line indicates median and scale bar in representative images are equivalent to 10 µm. Source data are provided as a Source Data file.

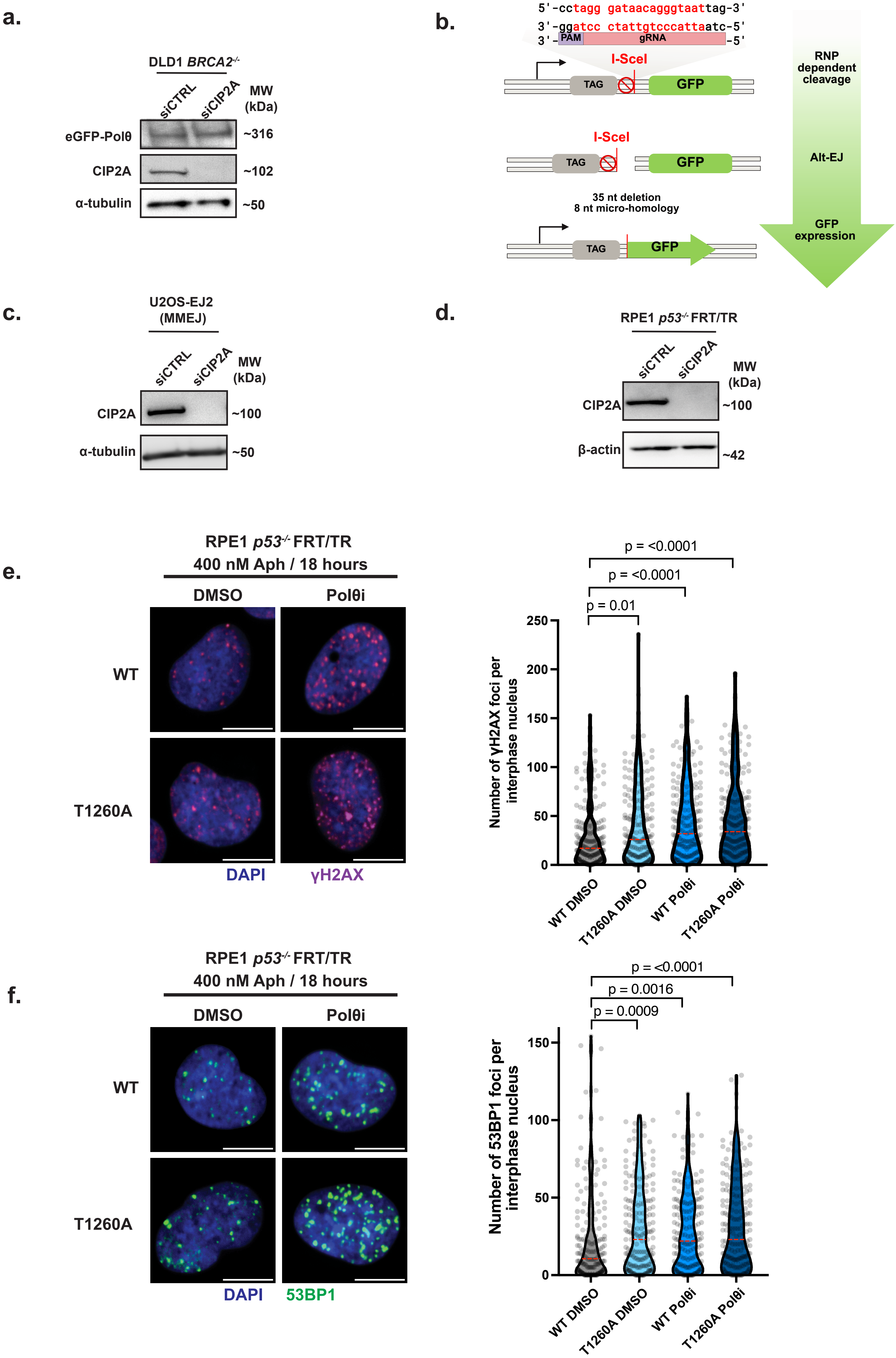
a.
DLD1 BRCA2-/-
siCTRL
siCIP2A
MW (kDa)
eGFP-Polθ
~316
CIP2A
~102
α-tubulin
~50
b.
5'-cctagg gataacagggtaattag-3'
3'-ggatcc ctattgtcccattaatc-5'
3'- PAM gRNA -5'
I-SceI
TAG
GFP
35 nt deletion
8 nt micro-homology
RNP dependent cleavage
Alt-EJ
GFP expression
c.
U2OS-EJ2 (MMEJ)
siCTRL
siCIP2A
MW (kDa)
CIP2A
~100
α-tubulin
~50
d.
RPE1 p53-/- FRT/TR
siCTRL
siCIP2A
MW (kDa)
CIP2A
~100
β-actin
~42
e.
RPE1 p53-/- FRT/TR
400 nM Aph / 18 hours
DMSO
Polθi
WT
T1260A
DAPI
γH2AX
p = <0.0001
p = <0.0001
p = 0.01
Number of γH2AX foci per interphase nucleus
0
50
100
150
200
250
WT DMSO
T1260A DMSO
WT Polθi
T1260A Polθi
f.
RPE1 p53-/- FRT/TR
400 nM Aph / 18 hours
DMSO
Polθi
WT
T1260A
DAPI
53BP1
p = <0.0001
p = 0.0016
p = 0.0009
Number of 53BP1 foci per interphase nucleus
0
50
100
150
WT DMSO
T1260A DMSO
WT Polθi
T1260A Polθi

Supplementary Figure 14

(a) Western blot analysis of eGFP- Polθ and CIP2A in DLD1 *BRCA2*$^{-/-}$ cells treated with siCTRL or siCIP2A followed by 100 ng doxycycline for 24 hours to induce eGFP- Polθ expression. (b) Schematic of the EJ2 Alt-EJ/MMEJ reporter system (Created in BioRender. Martin, P. (2025) https://BioRender.com/w53u255). (c) Western blot analysis of CIP2A in U2OS EJ2 reporter cells treated with siCTRL or siCIP2A. (d) Western blot analysis of CIP2A in RPE1 *p53*$^{-/-}$ FRT/TR cells treated with siCTRL or siCIP2A. (e). Representative images and violin plots of the number of γH2AX foci per interphase nucleus of RPE1 *p53*$^{-/-}$ FRT/TR WT or T1260A cells treated with DMSO or 5 μM ART558 (Polθi) and 400 nM aphidicolin for 18 hours (WT DMSO: n= 201, WT Polθi: n=201, T1260A DMSO: n= 201, T1260A Polθi: n= 201 from two independent experiments, statistical significance was determined by Mann-Whitney test). (f) Representative images and violin plots of the number of 53BP1 foci per interphase nucleus of RPE1 *p53*$^{-/-}$ FRT/TR WT or T1260A cells treated with DMSO or 5 μM ART558 (Polθi) and 400 nM aphidicolin for 18 hours (WT DMSO: n= 200, WT Polθi: n=200, T1260A DMSO: n= 200, T1260A Polθi: n= 200 from two independent experiments, statistical significance was determined by Mann-Whitney test). In e and f red line indicates medians, grey dots indicate individual values, representative image scale bars are equivalent to 10 μm. Source data are provided as a Source Data file.

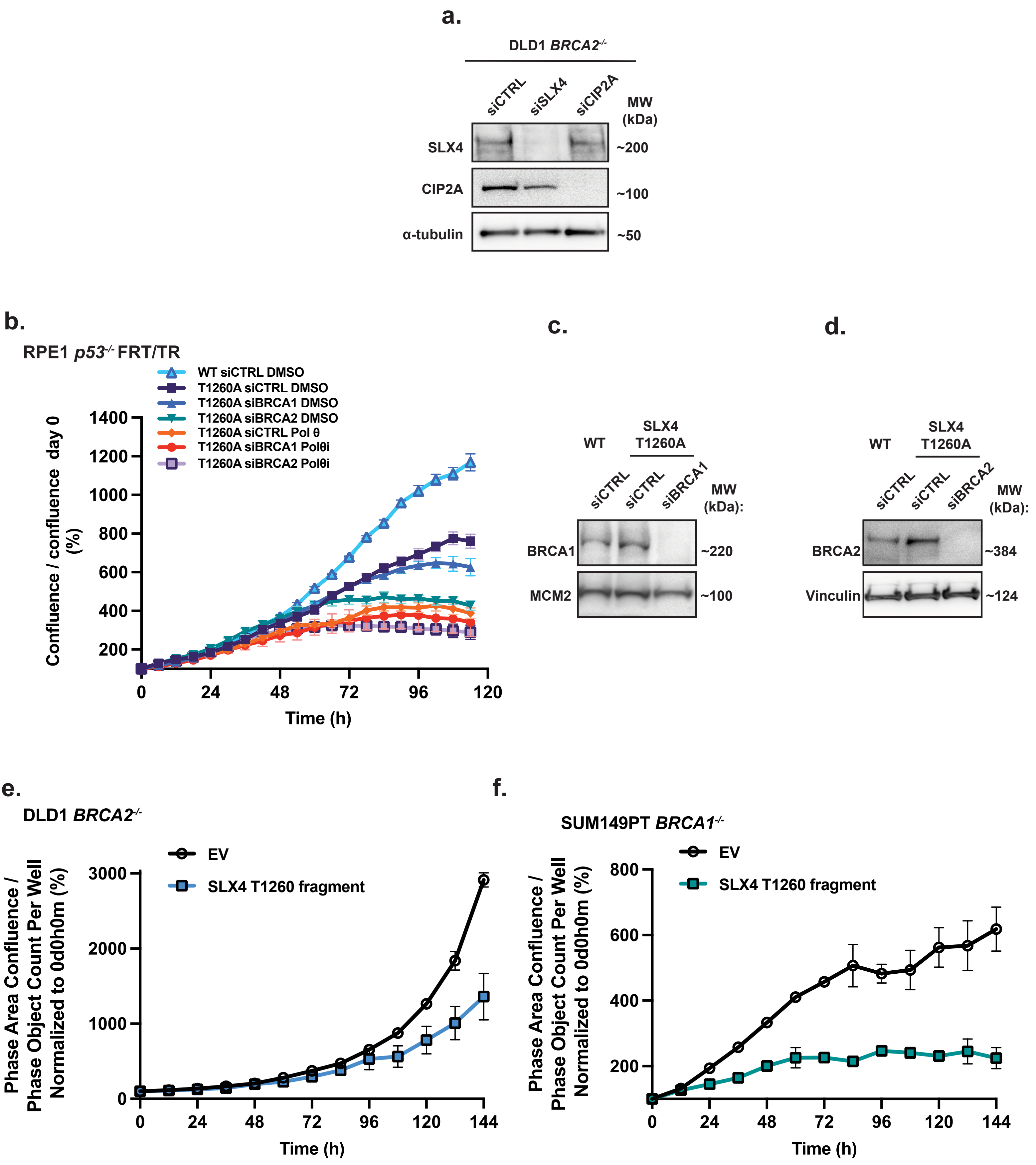
a.
DLD1 BRCA2-/-
siCTRL
siSLX4
siCIP2A
MW (kDa)
SLX4
~200
CIP2A
~100
α-tubulin
~50
b.
RPE1 p53-/- FRT/TR
WT siCTRL DMSO
T1260A siCTRL DMSO
T1260A siBRCA1 DMSO
T1260A siBRCA2 DMSO
T1260A siCTRL Pol θ
T1260A siBRCA1 Polθi
T1260A siBRCA2 Polθi
Confluence / confluence day 0 (%)
1400
1200
1000
800
600
400
200
0
24
48
72
96
120
Time (h)
c.
WT
SLX4 T1260A
siCTRL
siCTRL
siBRCA1
MW (kDa):
BRCA1
~220
MCM2
~100
d.
WT
SLX4 T1260A
siCTRL
siCTRL
siBRCA2
MW (kDa):
BRCA2
~384
Vinculin
~124
e.
DLD1 BRCA2-/-
EV
SLX4 T1260 fragment
Phase Area Confluence / Phase Object Count Per Well Normalized to 0d0h0m (%)
3000
2000
1000
0
0
24
48
72
96
120
144
Time (h)
f.
SUM149PT BRCA1-/-
EV
SLX4 T1260 fragment
Phase Area Confluence / Phase Object Count Per Well Normalized to 0d0h0m (%)
800
600
400
200
0
24
48
72
96
120
144
Time (h)

Supplementary Figure 15
(a) Western blot analysis of SLX4 and CIP2A in DLD1 *BRCA2*$^{-/-}$ cells treated with siCTRL, siSLX4 or siCIP2A. (b) Proliferation analysis using the Incucyte SX5 live cell analysis system of RPE1 *p53*$^{-/-}$ FRT/TR WT or T1260A knock in cells treated with siCTRL, siBRCA1 or siBRCA2, in addition to DMSO or 5 µM ART558 (Polθi) (from two independent experiments, Data points represent mean of two independent experiments with error bars representing SEM). (c) Western blot analysis of BRCA1 in RPE1 *p53*$^{-/-}$ FRT/TR WT or SLX4 T1260A treated with siCTRL or siBRCA1. (d) Western blot analysis of BRCA2 in RPE1 *p53*$^{-/-}$ FRT/TR WT or SLX4 T1260A treated with siCTRL or siBRCA2. (e) Proliferation analysis using the Incucyte S3 live cell analysis system of DLD1 *BRCA2*$^{-/-}$ cells with inducible expression of empty vector (EV) or a SLX4 T1260 fragment, incubated in the presence of 1 µg/ml doxycycline. Data points represent mean of two independent experiments with error bars representing SEM. (f) Proliferation analysis using the Incucyte S3 live cell analysis system of SUM149PT *BRCA1*$^{-/-}$ cells with inducible expression of empty vector (EV) or a SLX4 T1260 fragment, incubated in the presence of 1 µg/ml doxycycline. Data points represent mean of two independent experiments with error bars representing SEM. Source data are provided as a Source Data file.
