# Supplementary material for: The CIP2A-TOPBP1 axis facilitates mitotic DNA repair via MiDAS and MMEJ
Source: Nat Commun. 2025 Nov 27;16:10623. doi: 10.1038/s41467-025-65594-2 (PMC12661054; doi:10.1038/s41467-025-65594-2)
Supplement: Supplementary file 2 — Reporting Summary [file 41467_2025_65594_MOESM2_ESM.pdf]

nature portfolio

Corresponding author(s): Wojciech Niedzwiedz and Peter R Martin

Last updated by author(s): 21-09-25

Reporting Summary

Nature Portfolio wishes to improve the reproducibility of the work that we publish. This form provides structure for consistency and transparency in reporting. For further information on Nature Portfolio policies, see our Editorial Policies and the Editorial Policy Checklist.

Please do not complete any field with "not applicable" or n/a. Refer to the help text for what text to use if an item is not relevant to your study.
For final submission: please carefully check your responses for accuracy; you will not be able to make changes later.

Statistics

For all statistical analyses, confirm that the following items are present in the figure legend, table legend, main text, or Methods section.

n/a	Confirmed	
☐	☑	The exact sample size (n) for each experimental group/condition, given as a discrete number and unit of measurement
☐	☑	A statement on whether measurements were taken from distinct samples or whether the same sample was measured repeatedly
☐	☑	The statistical test(s) used AND whether they are one- or two-sided<br>*Only common tests should be described solely by name; describe more complex techniques in the Methods section.*
☑	☐	A description of all covariates tested
☑	☐	A description of any assumptions or corrections, such as tests of normality and adjustment for multiple comparisons
☐	☑	A full description of the statistical parameters including central tendency (e.g. means) or other basic estimates (e.g. regression coefficient) AND variation (e.g. standard deviation) or associated estimates of uncertainty (e.g. confidence intervals)
☐	☑	For null hypothesis testing, the test statistic (e.g. F, t, r) with confidence intervals, effect sizes, degrees of freedom and P value noted<br>*Give P values as exact values whenever suitable.*
☑	☐	For Bayesian analysis, information on the choice of priors and Markov chain Monte Carlo settings
☑	☐	For hierarchical and complex designs, identification of the appropriate level for tests and full reporting of outcomes
☑	☐	Estimates of effect sizes (e.g. Cohen's d, Pearson's r), indicating how they were calculated

Our web collection on statistics for biologists contains articles on many of the points above.

Software and code

Policy information about availability of computer code

Data collection	Fiji (ImageJ, RRID:SCR_003070), Cell Profiler (Broad Institute) and Aivia (Leica Microsystems), Graphpad Prism 10 (Dotmatics).
Data analysis	Fiji v2.16.0/1.54 (ImageJ, RRID:SCR_003070), Cell Profiler 2 v4.2. 6(Broad Institute) and Aivia v14.1.0 (Leica Microsystems), Graphpad Prism 10 V10.5.0 (Dotmatics)

For manuscripts utilizing custom algorithms or software that are central to the research but not yet described in published literature, software must be made available to editors and reviewers. We strongly encourage code deposition in a community repository (e.g. GitHub). See the Nature Portfolio guidelines for submitting code & software for further information.

Data

Policy information about availability of data

All manuscripts must include a data availability statement. This statement should provide the following information, where applicable:

- Accession codes, unique identifiers, or web links for publicly available datasets
- A description of any restrictions on data availability
- For clinical datasets or third party data, please ensure that the statement adheres to our policy

The data generated in this study are available within the article and its supplementary data files. The mass spectrometry proteomics data have been deposited to the ProteomeXchange Consortium via the PRIDE (10) partner repository with the dataset identifier PXD055936. Due to data size, microscopy imaging data is available upon request. Reagents generated in this study are also available upon request

Research involving human participants, their data, or biological material

Policy information about studies with human participants or human data. See also policy information about sex, gender (identity/presentation), and sexual orientation and race, ethnicity and racism.

Reporting on sex and gender	
Reporting on race, ethnicity, or other socially relevant groupings	
Population characteristics	
Recruitment	
Ethics oversight	

Note that full information on the approval of the study protocol must also be provided in the manuscript.

Field-specific reporting

Please select the one below that is the best fit for your research. If you are not sure, read the appropriate sections before making your selection.

☑ Life sciences ☐ Behavioural & social sciences ☐ Ecological, evolutionary & environmental sciences

For a reference copy of the document with all sections, see nature.com/documents/nr-reporting-summary-flat.pdf

Life sciences study design

All studies must disclose on these points even when the disclosure is negative.

Sample size	Included in figure legends.
Data exclusions	Data was not excluded
Replication	Included in figure legends.
Randomization	Microscopy images were captured and analysed in an unbiased manner and unbiased experimental design was implemented where possible. Where comparisons between control and treated samples was undertaken randomisation was not used.
Blinding	Where possible automation via software was used for quantitation. Blinding of data sets was implemented before analysis.

Behavioural & social sciences study design

All studies must disclose on these points even when the disclosure is negative.

Study description	
Research sample	
Sampling strategy	
Data collection	
Timing	
Data exclusions	
Non-participation	
Randomization	

Ecological, evolutionary & environmental sciences study design

All studies must disclose on these points even when the disclosure is negative.

Study description	
Research sample	
Sampling strategy	
Data collection	
Timing and spatial scale	
Data exclusions	
Reproducibility	
Randomization	
Blinding	

Did the study involve field work? ☐ Yes ☐ No

Field work, collection and transport

Field conditions	
Location	
Access & import/export	
Disturbance	

Reporting for specific materials, systems and methods

We require information from authors about some types of materials, experimental systems and methods used in many studies. Here, indicate whether each material, system or method listed is relevant to your study. If you are not sure if a list item applies to your research, read the appropriate section before selecting a response.

Materials & experimental systems

n/a	Involved in the study	
☐	☑	Antibodies
☐	☑	Eukaryotic cell lines
☑	☐	Palaeontology and archaeology
☑	☐	Animals and other organisms
☑	☐	Clinical data
☑	☐	Dual use research of concern
☑	☐	Plants

Methods

n/a	Involved in the study	
☑	☐	ChIP-seq
☐	☑	Flow cytometry
☑	☐	MRI-based neuroimaging

Antibodies

Antibodies used

For immunoblotting:
Primary antibodies: α-Tubulin (Sigma-Aldrich Cat# T5168, RRID:AB_477579, 1:100 000), β-Actin (Sigma-Aldrich Cat# A2066, RRID:AB_476693, 1:1000), MCM2 (Abcam Cat# ab4461, RRID:AB_304470 1:2000), H3 (Abcam Cat# ab1791, RRID:AB_302613, 1:50,000), Vinculin (Thermo Fisher Scientific Cat# MA5-11690, RRID:AB_10976821, 1:1000) GFP (Roche Cat# 11814460001, RRID:AB_390913, 1:500), MUS81 (Santa Cruz Biotechnology, (Santa Cruz Biotechnology Cat# sc-47692, RRID:AB_2147129,1:500), ERCC1 ((Santa Cruz Biotechnology Cat# sc-17809, RRID:AB_2278023,1:500), SLX4 (MRC-PPU Cat# S714C, RRID:AB_2752254, 1:500), SLX4 pT1260 (Genscript). TOPBP1 (Santa Cruz Biotechnology Cat# sc-271043, RRID:AB_10610636,1:500), MDC1 (Abcam Cat# ab11171, RRID:AB_297810, 1:1000), CIP2A (Santa Cruz Biotechnology Cat# sc-80659, RRID:AB_1121640 ,1:500), POLD3 (Abnova Cat# H00010714-M01, RRID:AB_606803, 1:500), TOP3A (Proteintech Cat# 14525-1-AP, RRID:AB_2205881, 1:3000), BLM (Bethyl Cat# A300-110A, RRID:AB_2064794, 1:500), BRCA1 (Millipore Cat# OP92, RRID:AB_2750876, 1:200), BRCA2 (Cell Signaling Technology Cat# 10741, RRID:AB_2797730, 1:1000).
Secondary antibodies:anti-mouse IgG-HRP (Dako, P0447, 1:2000), anti-rabbit IgG-HRP (Dako, P0448, 1:5000) and anti-sheep IgG-HRP (Abcam Cat# ab6747, RRID:AB_955453, 1:500).
For immunofluorescence:
Primary antibodies:mouse anti-MUS81 (Santa Cruz Biotechnology Cat# sc-53382, RRID:AB_2147138,1:500), mouse anti-ERCC1 (Santa Cruz Biotechnology Cat# sc-17809, RRID:AB_2278023, ,1:500), mouse anti-SLX4 (Abnova Cat# H00084464-B01P, RRID:AB_1673069, 1:500), mouse anti-TOPBP1 (Santa Cruz Biotechnology Cat# sc-271043, RRID:AB_10610636, 1:500), rabbit anti-TOPBP1 (Bethyl Cat# A300-111A, RRID:AB_2272050,1:500), mouse anti-MDC1 (Abcam Cat# ab50003, RRID:AB_881103, 1:500), mouse anti-CIP2A (Santa Cruz Biotechnology Cat# sc-80659, RRID:AB_1121640, 1:500), rabbit anti-CIP2A (Proteintech Cat# 23199-1-AP, RRID:AB_2918079,1:500), or GFP-Booster Alexa Fluor® 488 (ChromoTek Cat# gb2AF488-10, RRID:AB_2827573, 1:500) .
Secondary antibodies: used for immunofluorescence microscopy were: Alexa Fluor® 647 AffiniPure™ Donkey Anti-Mouse IgG (H+L) (Jackson ImmunoResearch Labs Cat# 715-605-150, RRID:AB_234086, 1:400), Donkey anti-Rabbit IgG (H+L) Highly Cross-Adsorbed Secondary Antibody, Alexa Fluor™ 647 (Molecular Probes Cat# A-31573, RRID:AB_2536183,1:400), Donkey anti-Mouse IgG (H+L) Highly Cross-Adsorbed Secondary Antibody, Alexa Fluor™ Plus 647 (Thermo Fisher Scientific Cat# A32787, RRID:AB_2762830, 1:500), Donkey anti-Mouse IgG (H+L) Highly Cross-Adsorbed Secondary Antibody, Alexa Fluor™ 488 (Molecular Probes Cat# A-21202, RRID:AB_141607, 1:400) or Donkey anti-Rabbit IgG (H+L) Highly Cross-Adsorbed Secondary Antibody, Alexa Fluor™ 488 (Thermo Fisher Scientific Cat# A-21206, RRID:AB_2535792, 1:400).

Validation

Use of previously validated antibodies or inclusion of siRNA/ or CRISPR knock out controls where possible. Use of antibodies that were also manufacuter validated and literature validated.

Eukaryotic cell lines

Policy information about cell lines and Sex and Gender in Research

Cell line source(s)

HCT116-TOPBP1-mAID-Clover provided by D. Cortez (Vanderbilt University); RPE1 p53-/- FRT/TR provided by S.P Jackson (University of Cambridge) RPE1 p53-/- PAC-/- WT and BRCA1-/- provided by S.M. Noodermeer (Leiden University Medical Centre); HEK293TN from Systems Biosciences; DLD1, DLD1 BRCA2-/- and SUM149PT BRCA1-/- from C. Lord (Institute of Cancer Research; U2OS pBIR from T. D. Halzonetis (University of Geneva); U2OS EJ2 from J. M. Stark (Beckman Research Institute of the City of Hope).

Authentication

HEK293TN cells were purchased from Systems Biosciences. Cell lines used in this study were provided by acknowledged source. Genetic interaction with CIP2A loss confirmed DLD1 and DLD1 BRCA2 deficient cell lines. HCT116-mAID-TOPBP1-Clover was confimed by degredation of TOPBP1. RPE1 p53-/- FRT/TR cells were confirmd by FLP in recombination. RPE1 p53-/- PAC-/- cells were validated by puromycin sensitivity. SUM149PT were previously STR profiled. Presence of transgene was confirmed by fluorescence microscopy and western blotting. Knock out cells were confirmed by western blot and knock in by RFLP and sanger

Mycoplasma contamination

Regular testing was carried out throughout the study and all populations analysed tested negative.

Commonly misidentified lines
(See ICLAC register)

None of the cell lines used are listed in the database of commonly misidentified cell lines.

Palaeontology and Archaeology

Specimen provenance

Specimen deposition

Dating methods

☐ Tick this box to confirm that the raw and calibrated dates are available in the paper or in Supplementary Information.

Ethics oversight

Note that full information on the approval of the study protocol must also be provided in the manuscript.

Animals and other research organisms

Policy information about studies involving animals; ARRIVE guidelines recommended for reporting animal research, and Sex and Gender in Research

Laboratory animals

Wild animals

Reporting on sex

Field-collected samples

Ethics oversight

Note that full information on the approval of the study protocol must also be provided in the manuscript.

Clinical data

Policy information about clinical studies

All manuscripts should comply with the ICMJE guidelines for publication of clinical research and a completed CONSORT checklist must be included with all submissions.

Clinical trial registration

Study protocol

Data collection

Outcomes

Dual use research of concern

Policy information about dual use research of concern

Hazards

Could the accidental, deliberate or reckless misuse of agents or technologies generated in the work, or the application of information presented in the manuscript, pose a threat to:

No | Yes

- [ ] [ ] Public health
- [ ] [ ] National security
- [ ] [ ] Crops and/or livestock
- [ ] [ ] Ecosystems
- [ ] [ ] Any other significant area

Experiments of concern

Does the work involve any of these experiments of concern:

No | Yes

- [ ] [ ] Demonstrate how to render a vaccine ineffective
- [ ] [ ] Confer resistance to therapeutically useful antibiotics or antiviral agents
- [ ] [ ] Enhance the virulence of a pathogen or render a nonpathogen virulent
- [ ] [ ] Increase transmissibility of a pathogen
- [ ] [ ] Alter the host range of a pathogen
- [ ] [ ] Enable evasion of diagnostic/detection modalities
- [ ] [ ] Enable the weaponization of a biological agent or toxin
- [ ] [ ] Any other potentially harmful combination of experiments and agents

Plants

Seed stocks

Novel plant genotypes

Authentication

ChIP-seq

Data deposition

- [ ] Confirm that both raw and final processed data have been deposited in a public database such as GEO.
- [ ] Confirm that you have deposited or provided access to graph files (e.g. BED files) for the called peaks.

Data access links
May remain private before publication.

Files in database submission

Genome browser session
(e.g. UCSC)

Methodology

Replicates

Sequencing depth

Antibodies

Peak calling parameters

Data quality

Software

Flow Cytometry

Plots

Confirm that:

- [x] The axis labels state the marker and fluorochrome used (e.g. CD4-FITC).
- [x] The axis scales are clearly visible. Include numbers along axes only for bottom left plot of group (a 'group' is an analysis of identical markers).
- [ ] All plots are contour plots with outliers or pseudocolor plots.
- [x] A numerical value for number of cells or percentage (with statistics) is provided.

Methodology

Sample preparation: Included in material and methods.

Instrument: Included in material and methods.

Software: Included in material and methods.

Cell population abundance: Included in material and methods.

Gating strategy: Included in material and methods.

- [ ] Tick this box to confirm that a figure exemplifying the gating strategy is provided in the Supplementary Information.

Magnetic resonance imaging

Experimental design

Design type

Design specifications

Behavioral performance measures

Imaging type(s)

Field strength

Sequence & imaging parameters

Area of acquisition

Diffusion MRI ☐ Used ☐ Not used

Preprocessing

Preprocessing software

Normalization

Normalization template

Noise and artifact removal

Volume censoring

Statistical modeling & inference

Model type and settings

Effect(s) tested

Specify type of analysis: ☐ Whole brain ☐ ROI-based ☐ Both

Statistic type for inference

(See Eklund et al. 2016)

Correction

Models & analysis

n/a	Involved in the study
☐	☐ Functional and/or effective connectivity
☐	☐ Graph analysis
☐	☐ Multivariate modeling or predictive analysis

Functional and/or effective connectivity

Graph analysis

Multivariate modeling and predictive analysis

This checklist template is licensed under a Creative Commons Attribution 4.0 International License, which permits use, sharing, adaptation, distribution and reproduction in any medium or format, as long as you give appropriate credit to the original author(s) and the source, provide a link to the Creative Commons license, and indicate if changes were made. The images or other third party material in this article are included in the article's Creative Commons license, unless indicated otherwise in a credit line to the material. If material is not included in the article's Creative Commons license and your intended use is not permitted by statutory regulation or exceeds the permitted use, you will need to obtain permission directly from the copyright holder. To view a copy of this license, visit http://creativecommons.org/licenses/by/4.0/
