# Supplementary material for: The CIP2A-TOPBP1 axis facilitates mitotic DNA repair via MiDAS and MMEJ
Source: Nat Commun. 2025 Nov 27;16:10623. doi: 10.1038/s41467-025-65594-2 (PMC12661054; doi:10.1038/s41467-025-65594-2)

Supplementary Figure 1d

Membrane was cut at ~ 70 kDA marker and lower portion incubated with
anti tubulin and upper portion incubated with anti TOPBP1. Membranes were processed and developed in parallel.

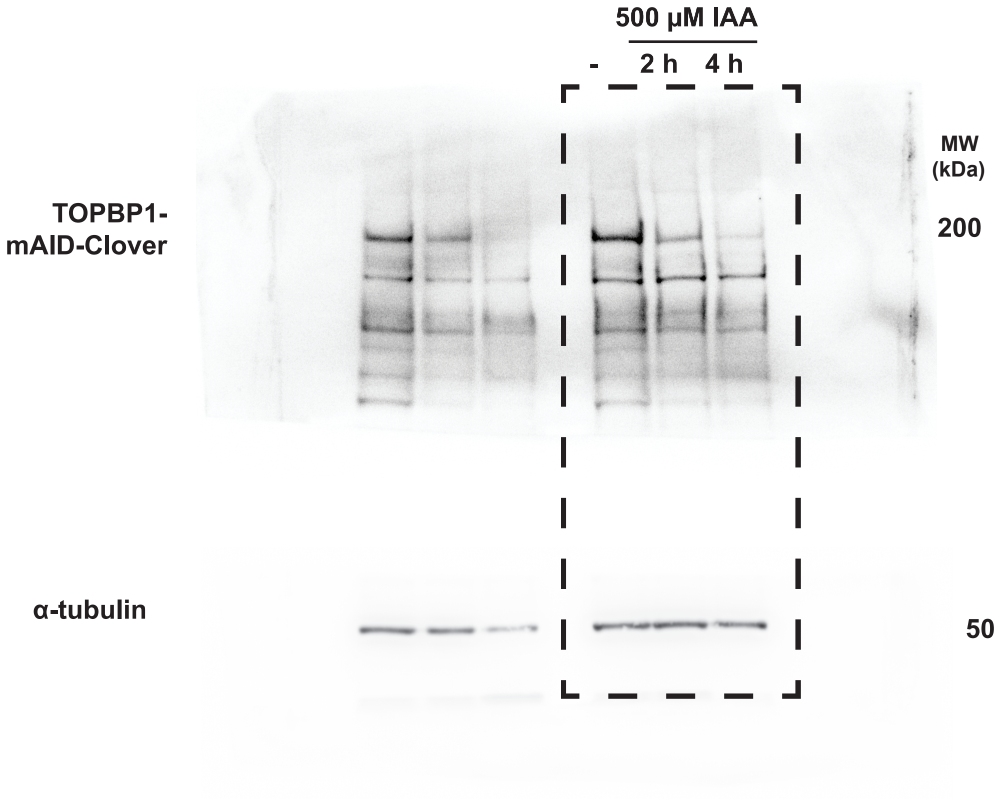

Supplementary Figure 1e.
Uncropped membranes

10 % of Input and IP volume was ran independently on separate gels and blots for detection of bait only.
90% of Input and IP volume was ran independently on separate gels and blots.

Detection of interacting proteins on the same membrane by cutting of membrane horizontally
and independent incubation with primary antibodies.

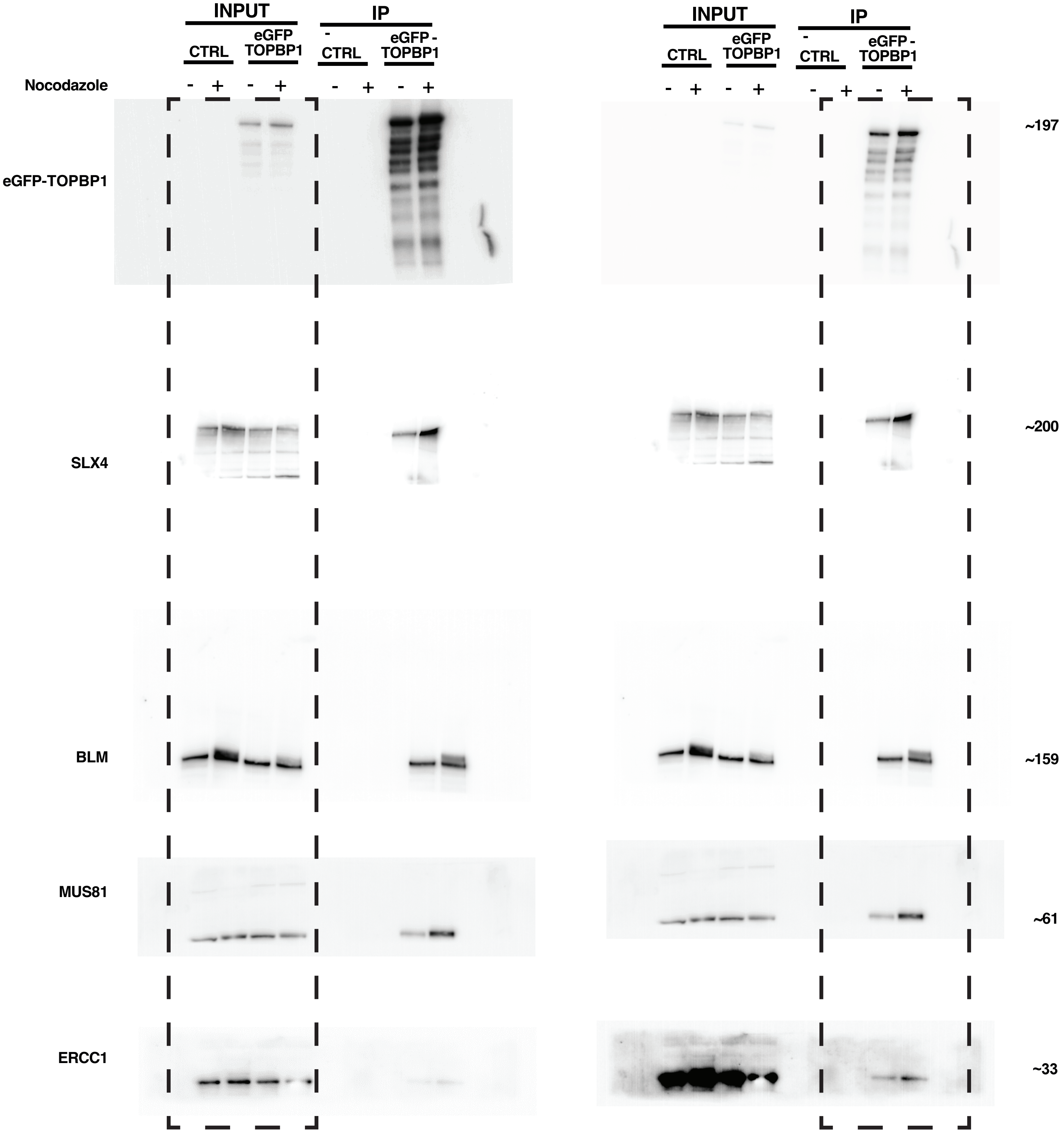

Supplementary Figure 2g, and i
Membranes were cut horizontally to seperate RNAi target protein molecular weight from tubulin loading control. All membrane pieces were incubated in parallel with respective primary antibodies and developed in parallel.

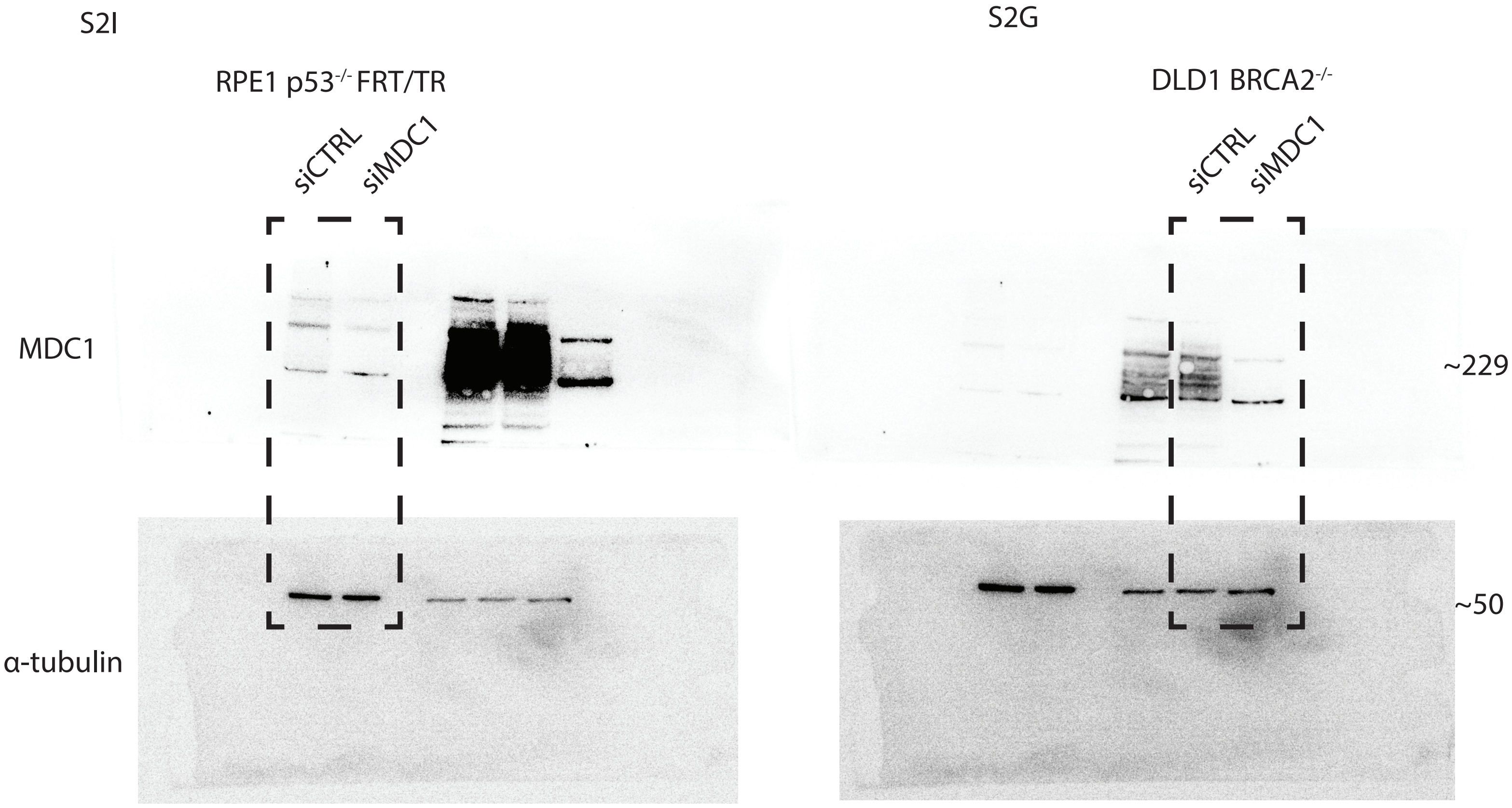

Supplementary Figures 2h, S6d and f
Membranes were cut horizontally to seperate RNAi target protein molecular weight from tubulin loading control, cut vertically at marker positions to separate RNAi treated samples. All membrane pieces were incubated in parallel with respective primary antibodies and developed in parallel.

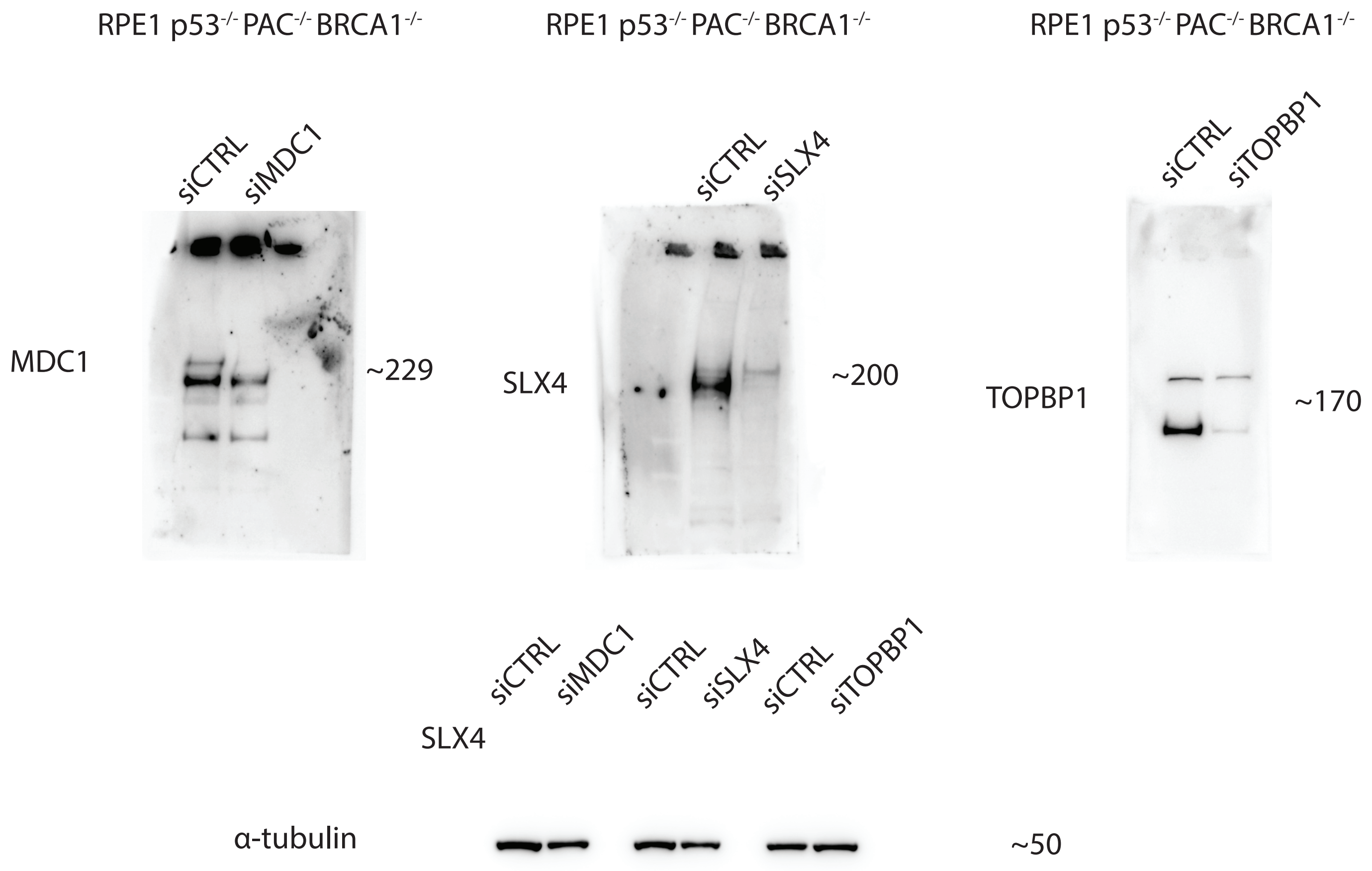

Figure 3b and Supplementary Figure 7a
Uncropped membranes

10 % of Input and IP volume was ran independently on sepearate gels and blots for detection of bait only.
90% of Input and IP volume was ran independently on sepearate gels and blots.

Detection of interacting proteins on the same membrane by cutting of membrane
and independent incubation with primary antibodies.

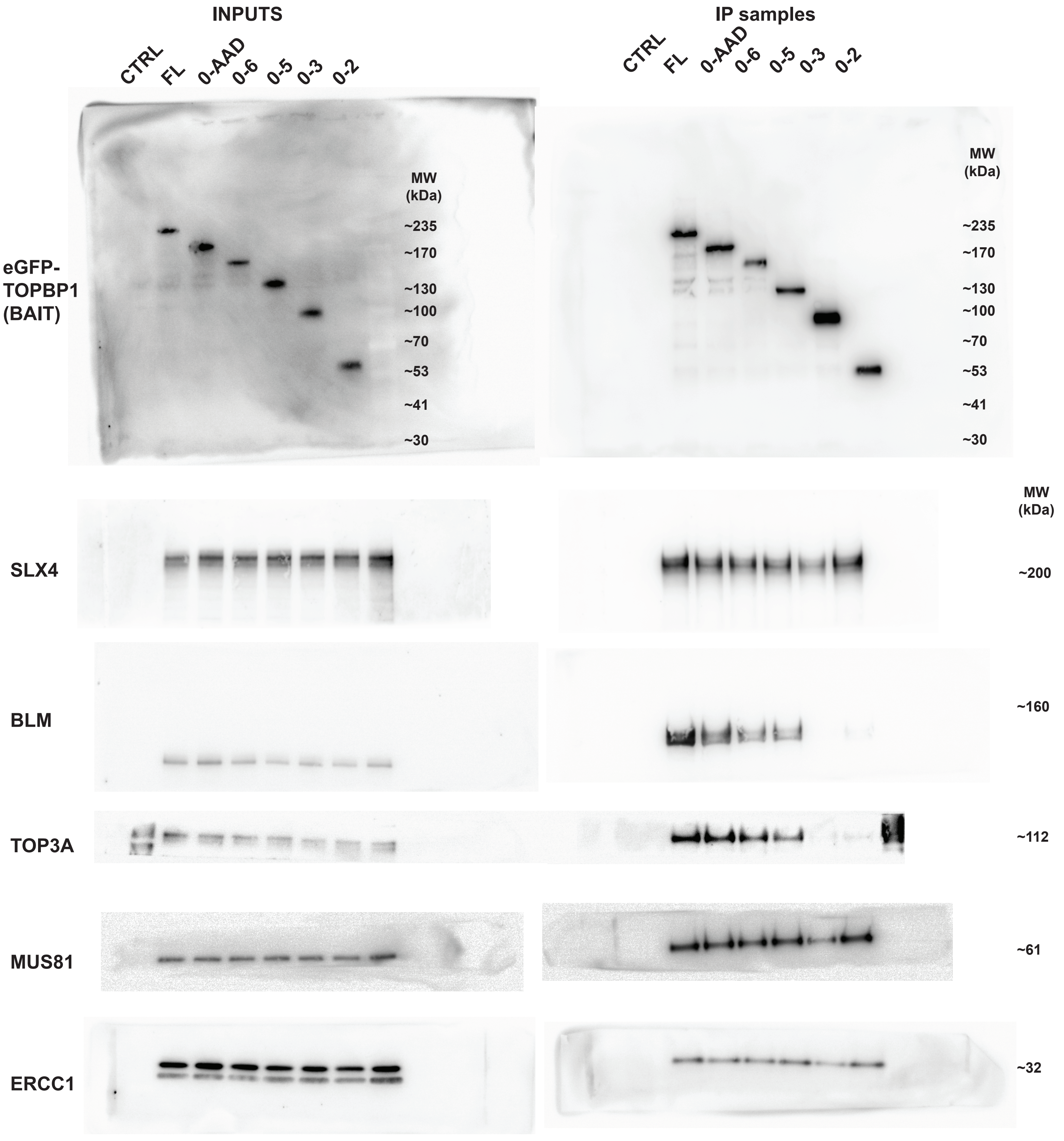

Figure 3c
Uncropped membranes

10 % of Input and IP volume was ran independently on separate gels and blots for detection of bait only.
90% of Input and IP volume was ran independently on separate gels and blots.

Detection of interacting proteins on the same membrane by cutting of membrane.

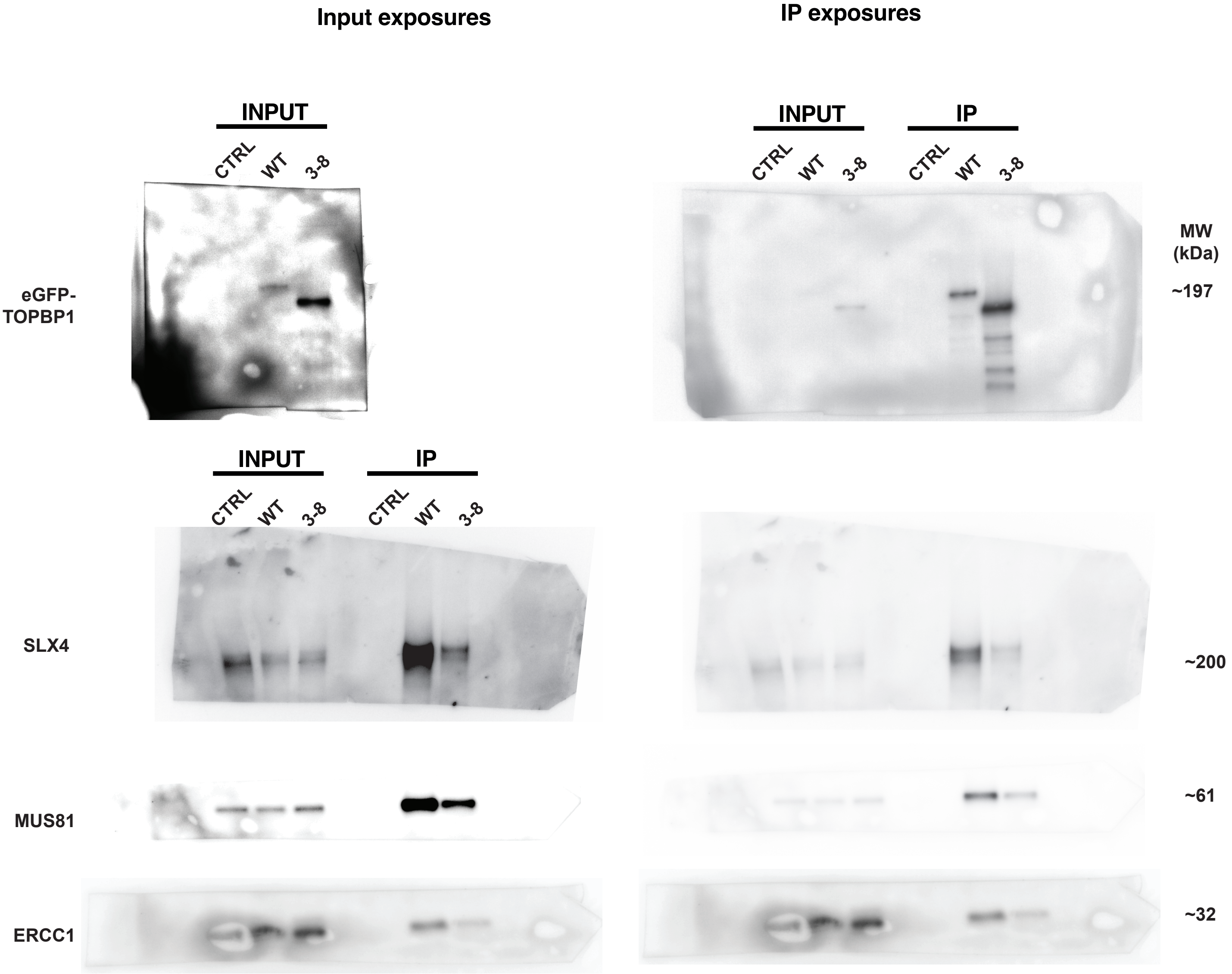

Figure 4a
Input samples were ran independently in duplicate to demonstrate successful fractionation (Left panel). Chromatin Input and 50% of chromatin IP was ran in duplicate on independent gels and membranes, Membranes were cut horizontally and incubated with respective primary antibodies and then developed in parallel.

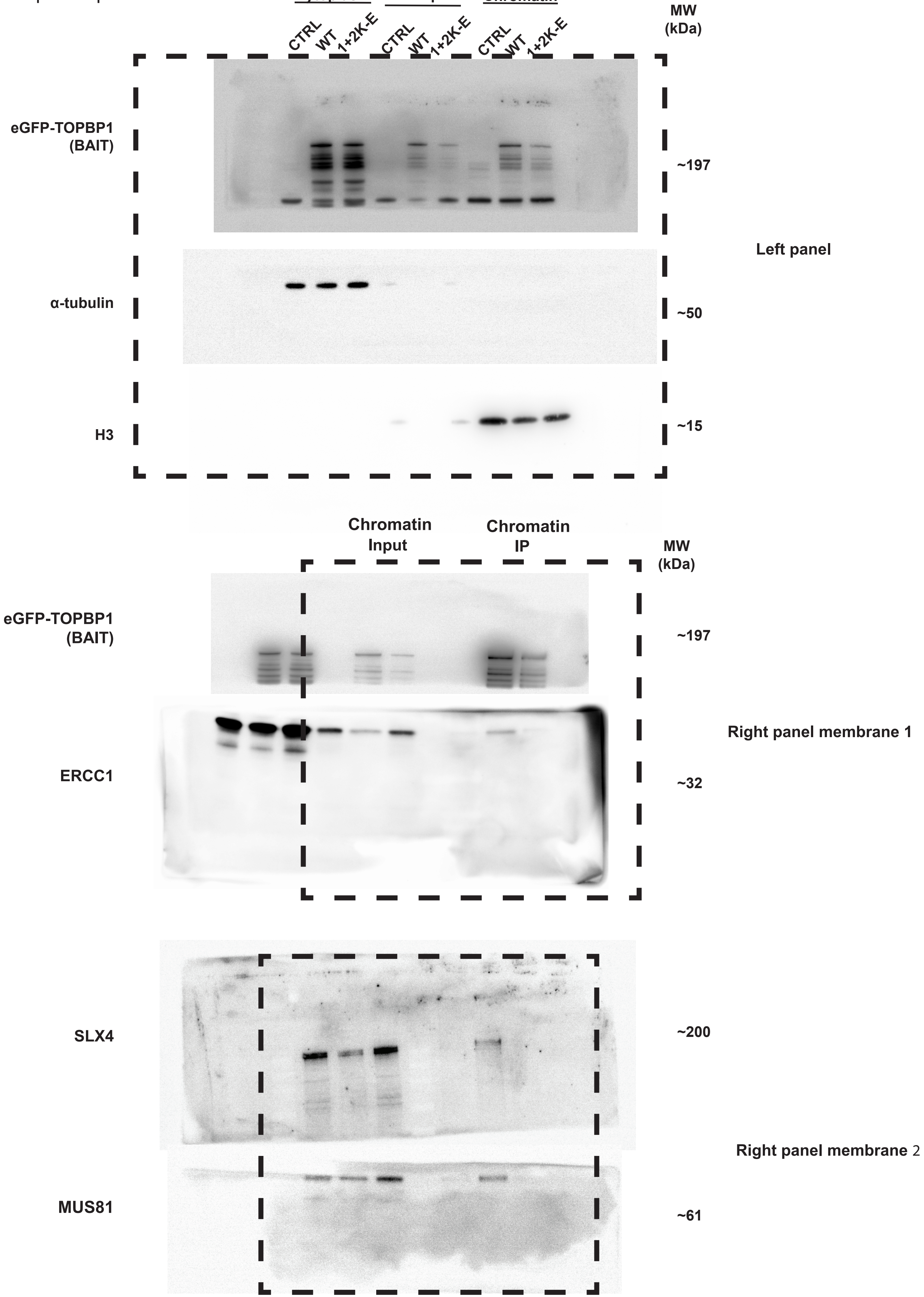

Figure 4b
Uncropped membranes

10 % of Input and IP volume was ran independently on separate gels and blots for detection of bait only.
90% of Input and IP volume was ran independently on separate gels and blots.

Detection of interacting proteins on the same membrane by cutting of membrane
and independent incubation with primary antibodies.

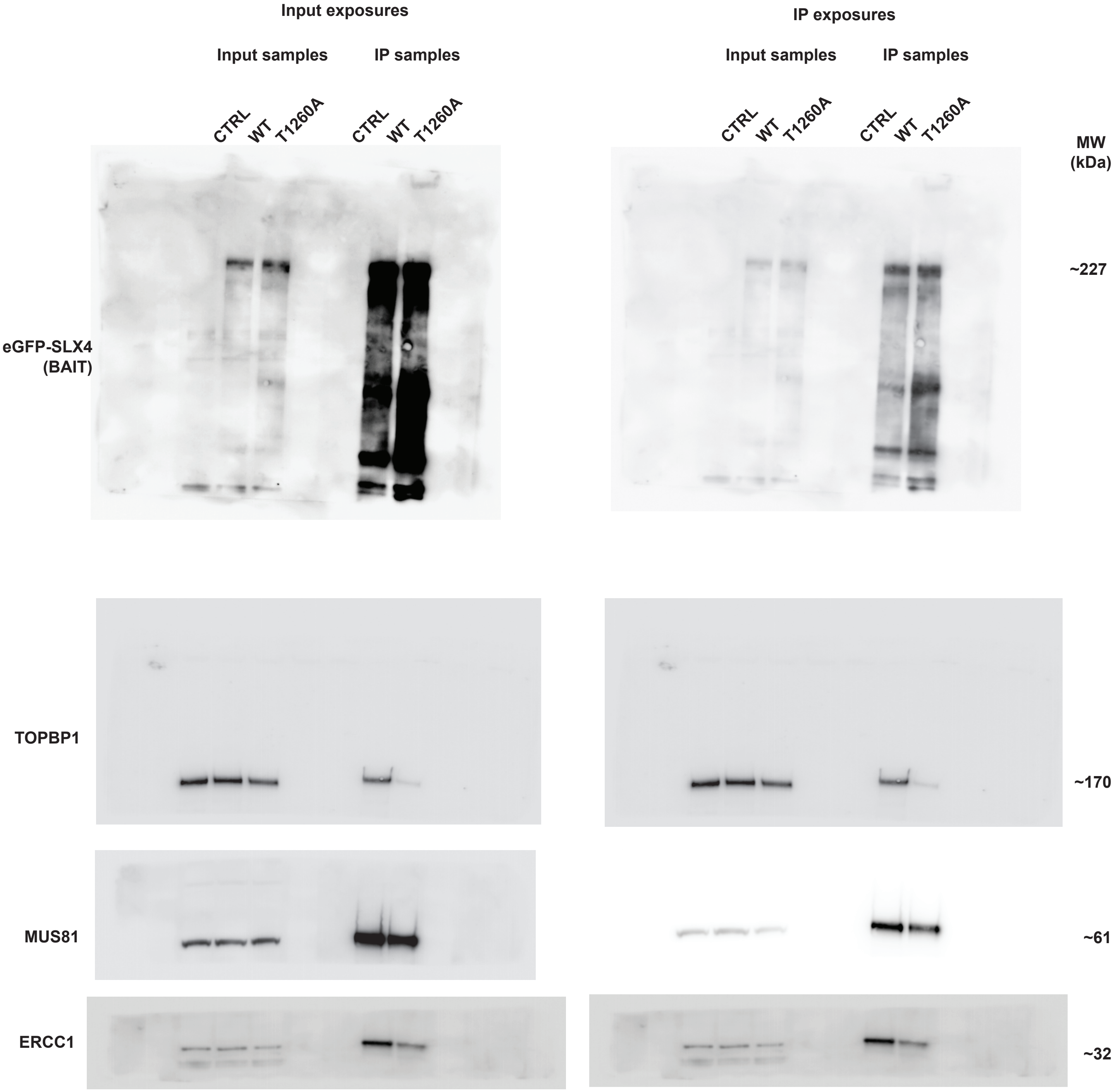

Figure 4c
Uncropped membranes

10 % of Input and IP volume was ran independently on separate gels and blots for detection of bait only.
90% of Input and IP volume was ran independently on separate gels and blots.

Detection of interacting proteins on the same membrane by cutting of membrane and independent incubation with primary antibodies. Total SLX4 was probed on the same membrane piece post pT1260 probing and development.

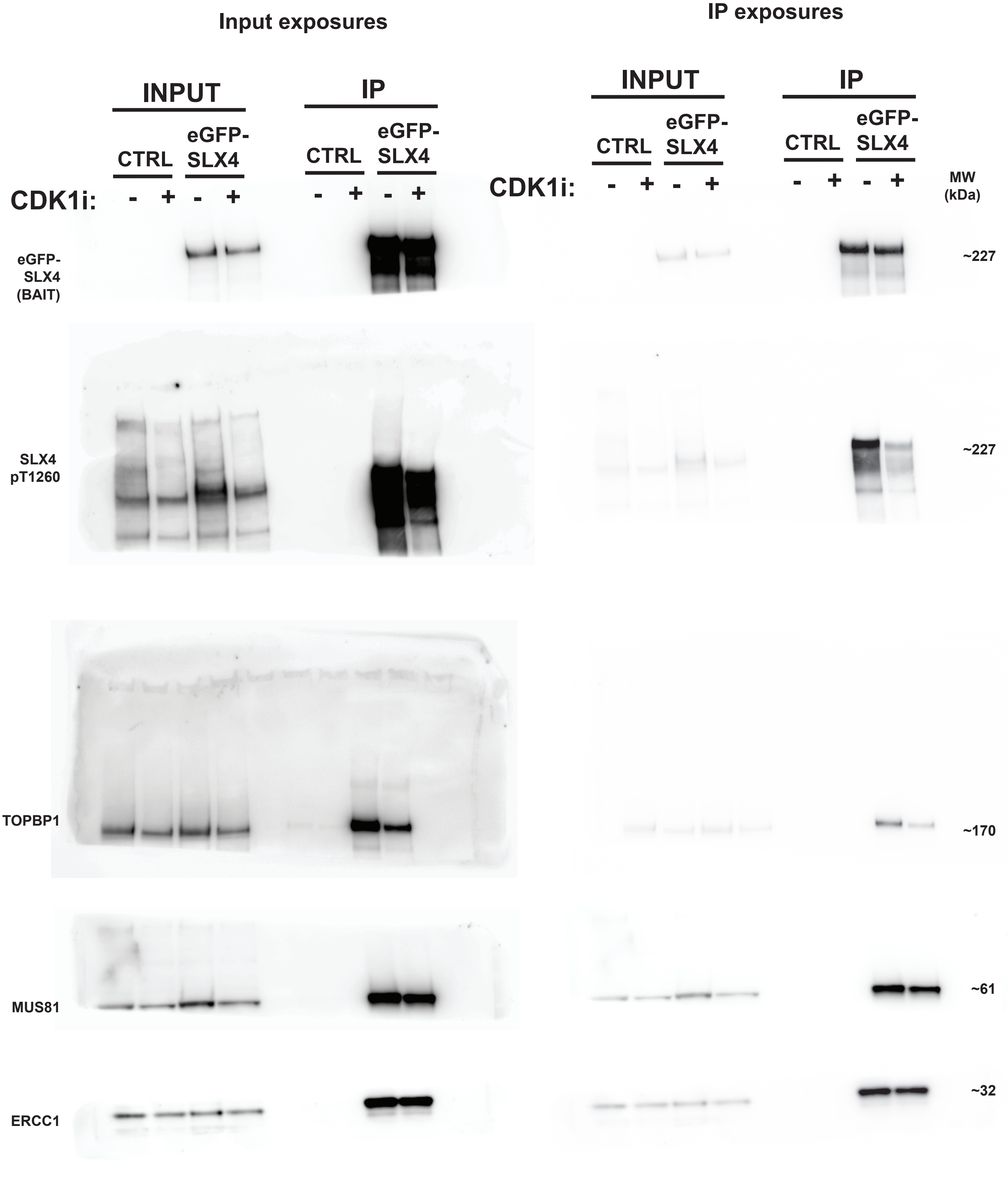

Figure 4d
Uncropped membranes

10 % of Input and IP volume was ran independently on separate gels and blots for detection of bait only.
90% of Input and IP volume was ran independently on separate gels and blots.

Detection of interacting proteins on the same membrane by cutting of membrane
and independent incubation with primary antibodies. Total SLX4 was probed on the same membrane piece post pT1260 probing and development.

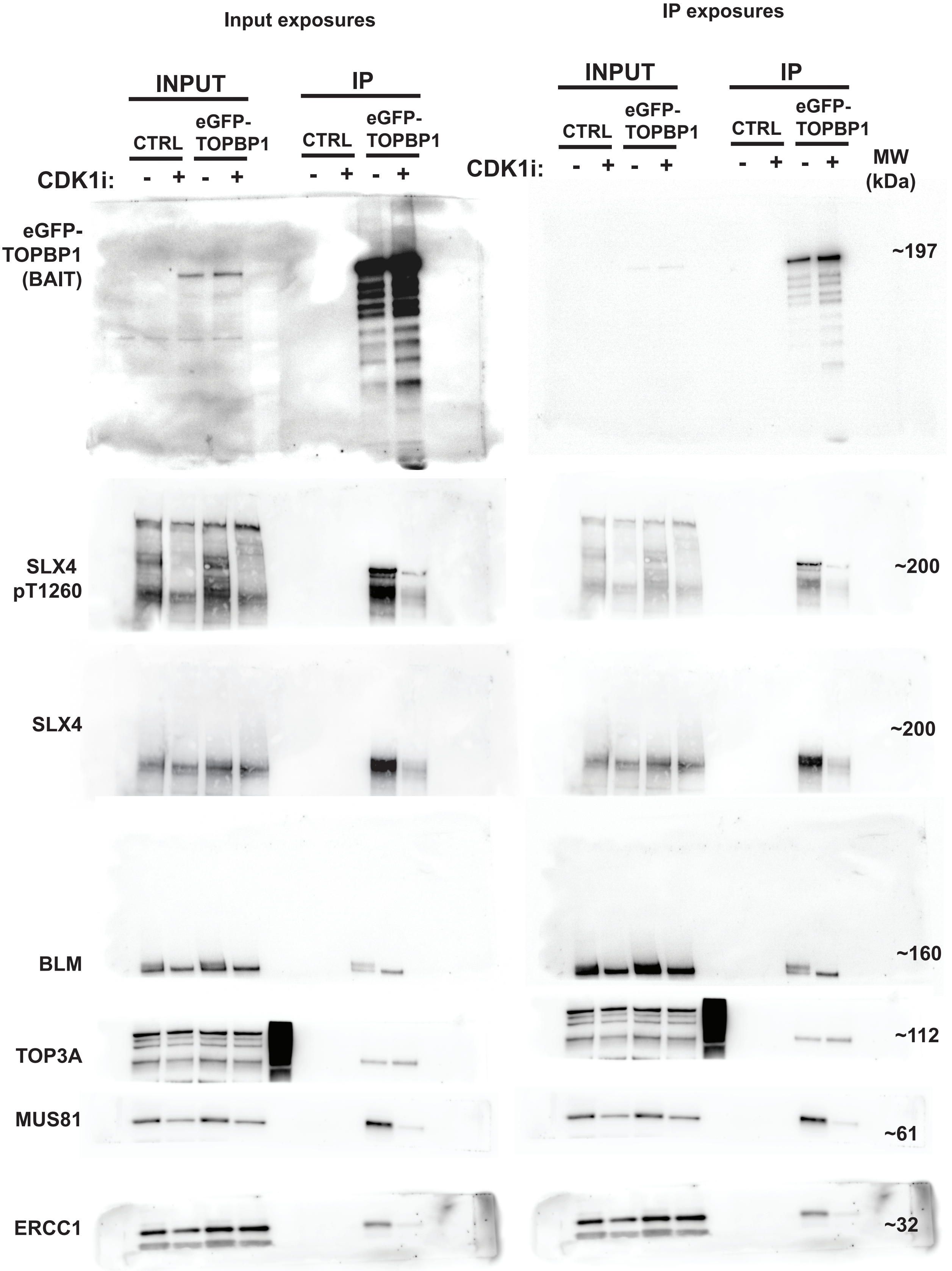

Supplementary Figure 6a

Membrane was cut at ~ 70 kDA marker and lower portion incubated with anti tubulin and upper portion incubated with anti TOPBP1. Membranes were processed and developed in parallel.

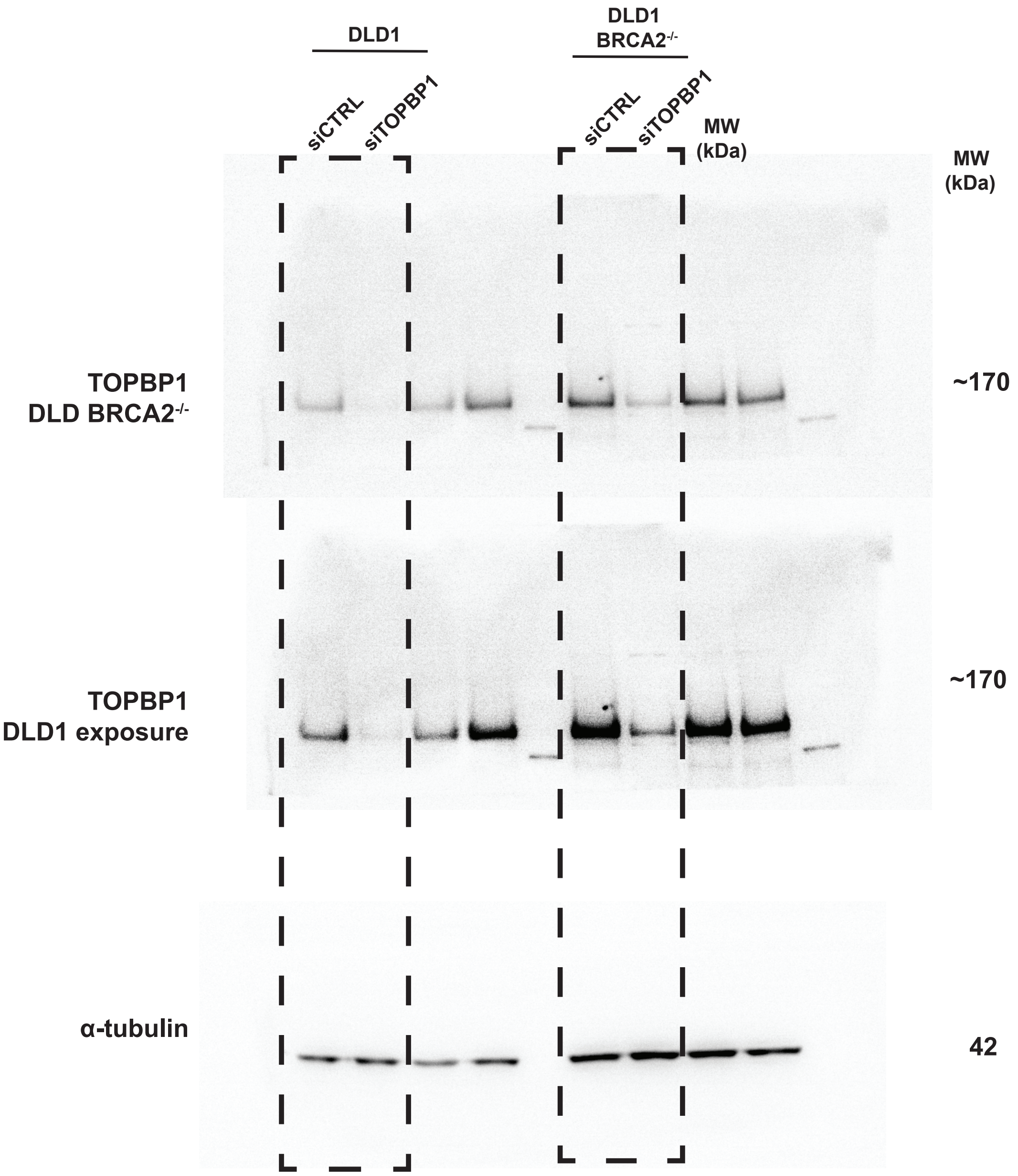

Supplementary Figure 6b

Membranes were cut horizontally and pieces were probed independently with antibodies. DLD1 and DLD1 BRCA2-/- cell line samples probed with anti SLX4 were divided by vertically cutting the membrane to enable longer exposure of DLD1 BRCA2$^{-/-}$ samples. Tubulin for both DLD1 and DLD1 BRCA2$^{-/-}$ was developed on the same membrane simultaneously.

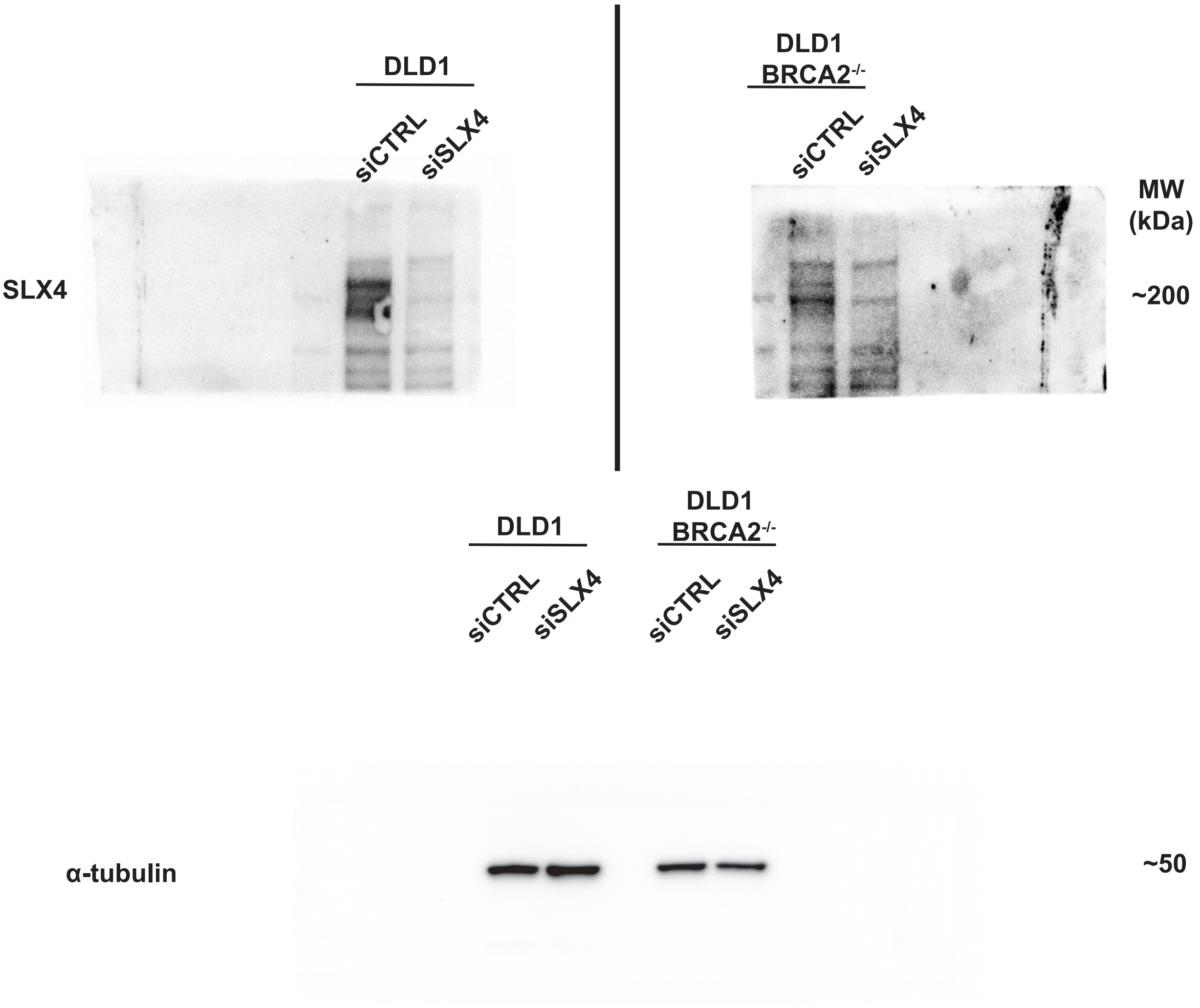

Supplementary Figure 8a
Uncropped membranes

10 % of Input and IP volume was ran independently on sepearate gels and blots for detection of bait only.
90% of Input and IP volume was ran independently on sepearate gels and blots.

Detection of interacting proteins on the same membrane by cutting of membrane
and independent incubation with primary antibodies.

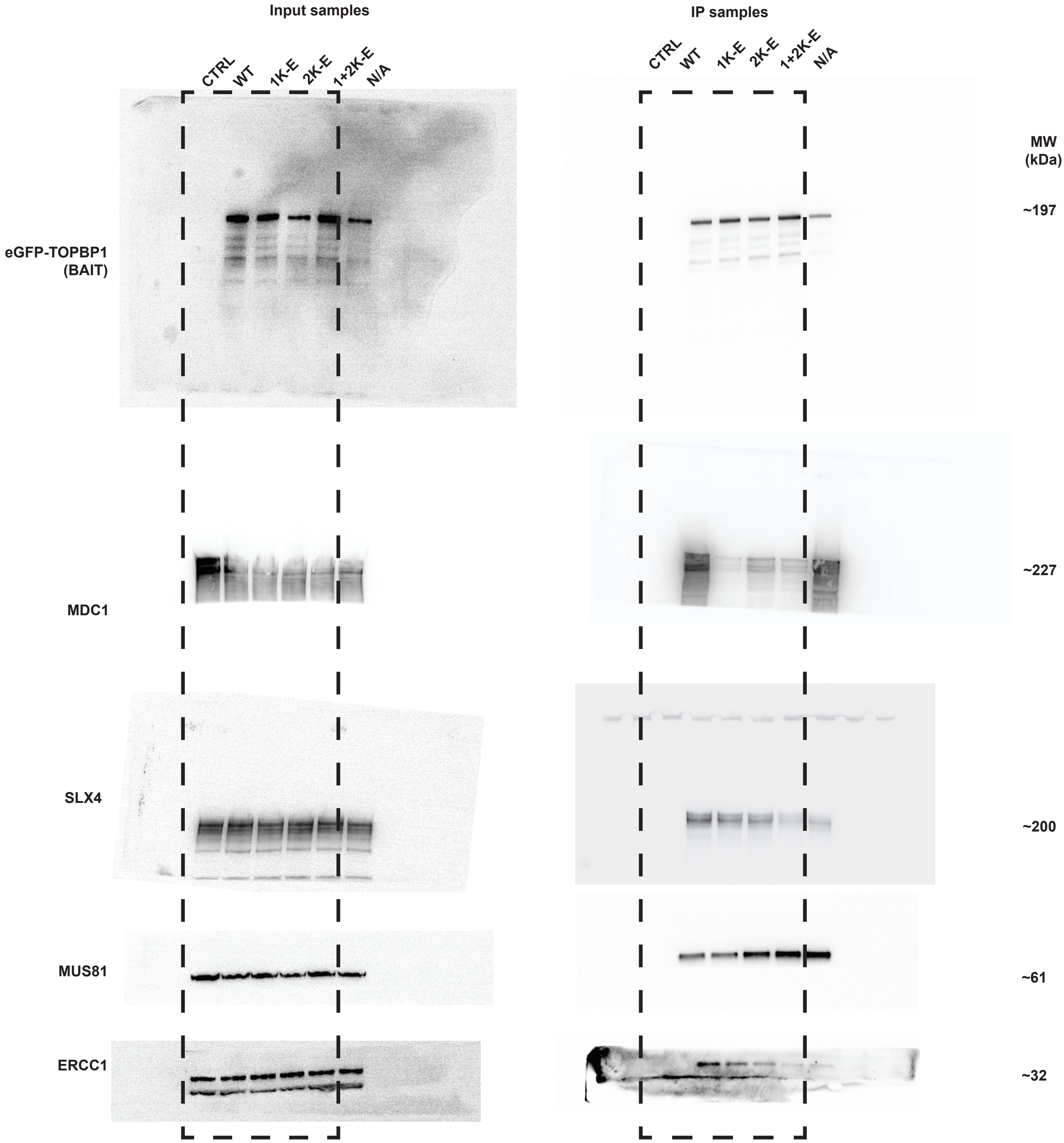

Supplementary Figure 8b

Membrane was cut at ~ 70 kDA marker and lower portion incubated with
anti tubulin and upper portion incubated with anti SLX4 pT1260, followed by SLX4.
Membranes were processed and developed in parallel.
SLX4 pT1260 was probed initially in the upper piece of the membrane
followed by reprobing with total SLX4 antibody.

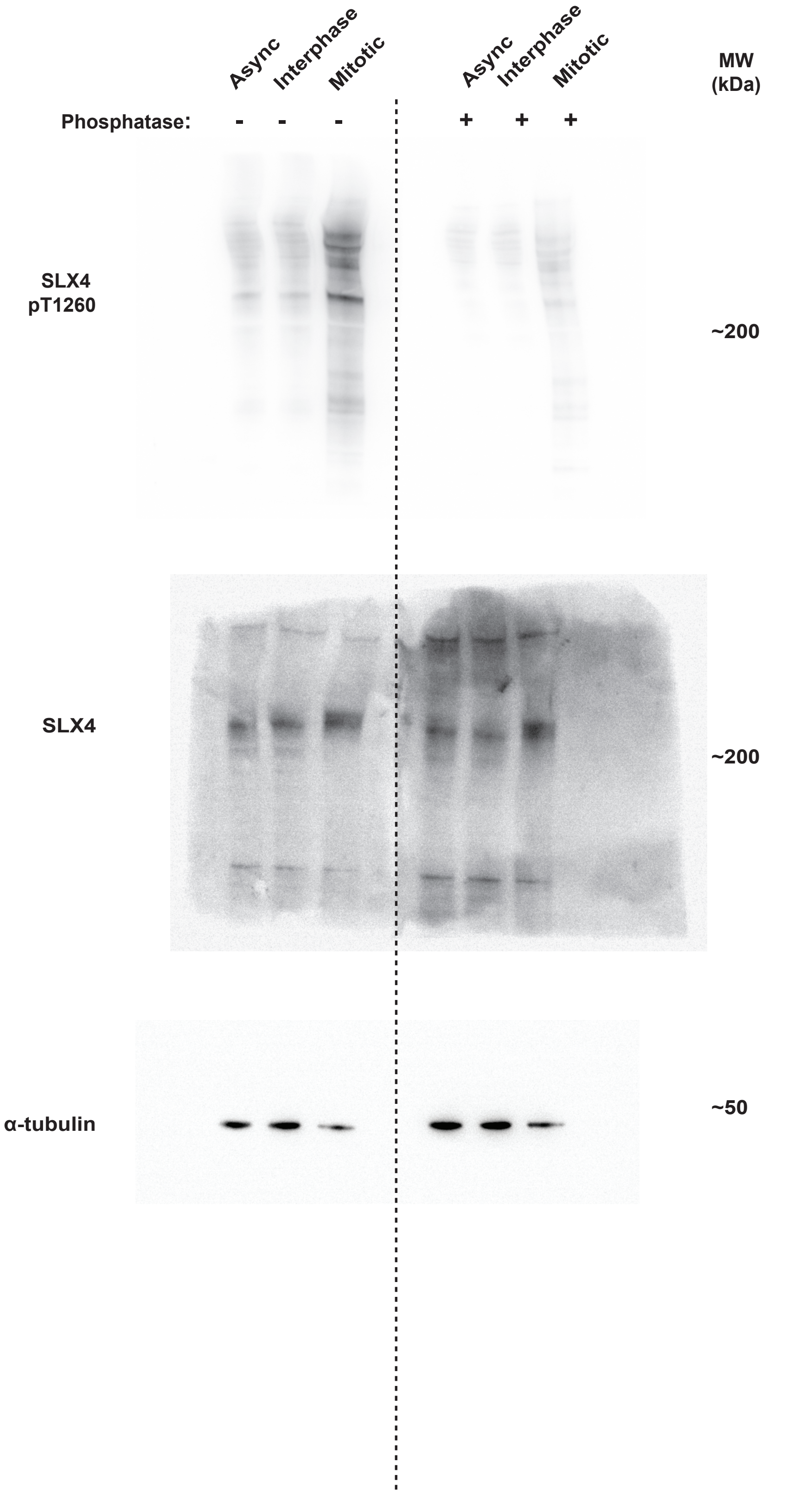

Supplementary Figure 8c

10% of cytoplasmic fraction and chromatin input were ran on an independent gel and membrane to confirm successful fractionation through probing and detecting tubulin and H3 respectively. A further 10% of the cytoplasmic fraction was ran on an independent gel and membrane to detect eGFP-SLX4 (BAIT) and MUS81 in the cytoplasmic fraction. A further 10% of the chromatin input amount of protein (10 μg of 100μg input to IP) was ran on an independent gel along side all of elute of the IP from the chromatin fraction. The membranes were divided so that they could be incubated with antibodies against the respective proteins and developed in parallel.

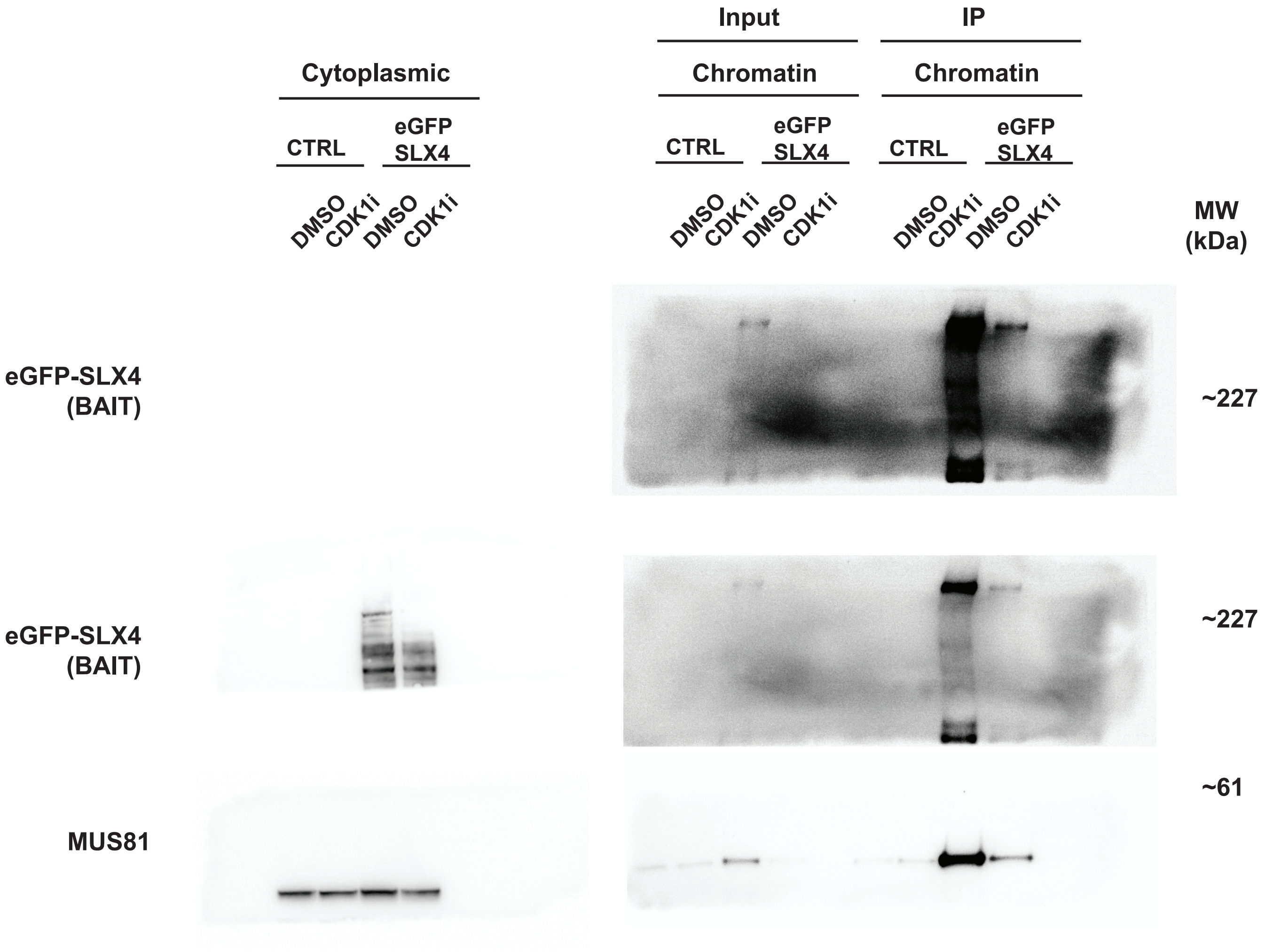

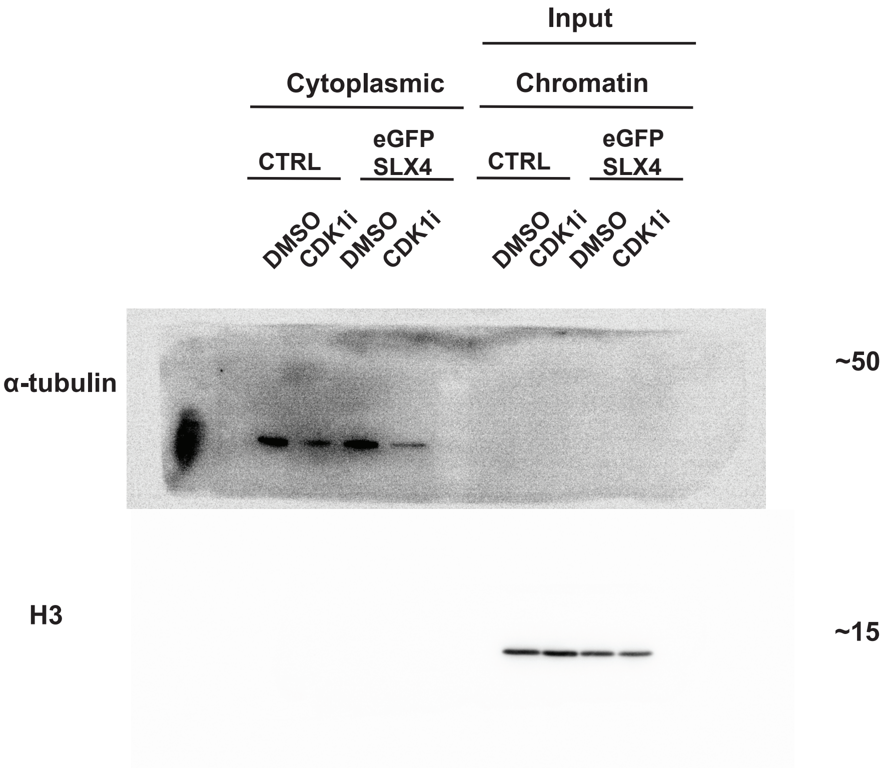

Supplementary figure 9a

Membranes were cut horizontally and pieces were probed independently with antibodies.

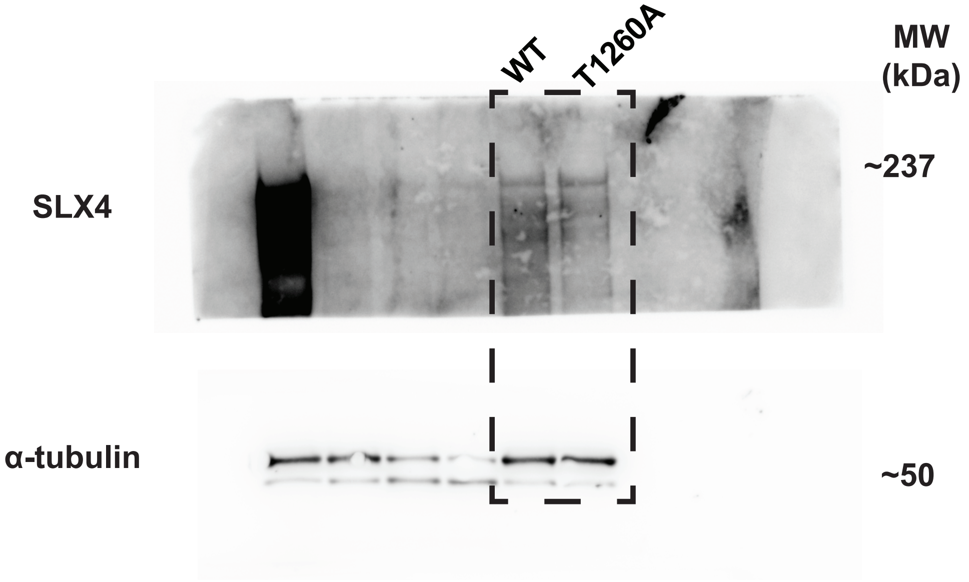

Supplementary Figure 10c

Membrane was cut at ~ 70 kDA marker and lower portion incubated with
anti tubulin and upper portion incubated with anti SLX4. Membranes were processed and developed in parallel.

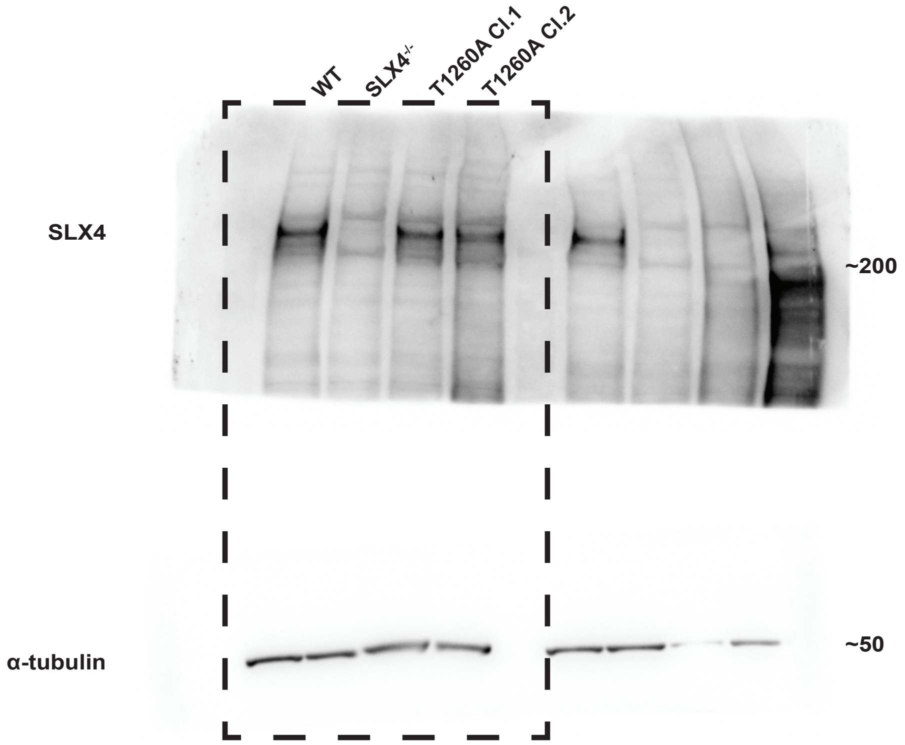

Supplementary Figure 11c

Membranes were cut horizontally and pieces were probed independently with antibodies.

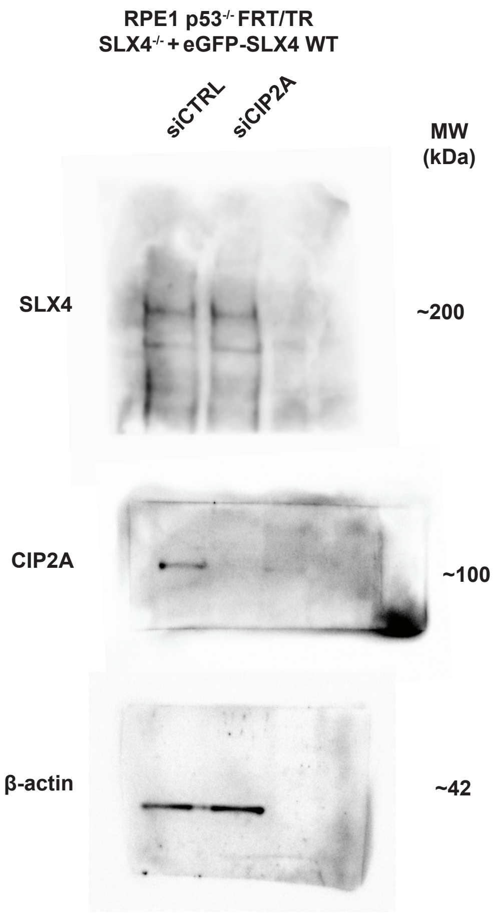

Supplementary Figure 13a
Membranes were cut horizontally to seperate molecular weight of CIP2A from tubulin loading control.
Membrane pieces were incubated in parallel with respective primary antibodies and developed in parallel.

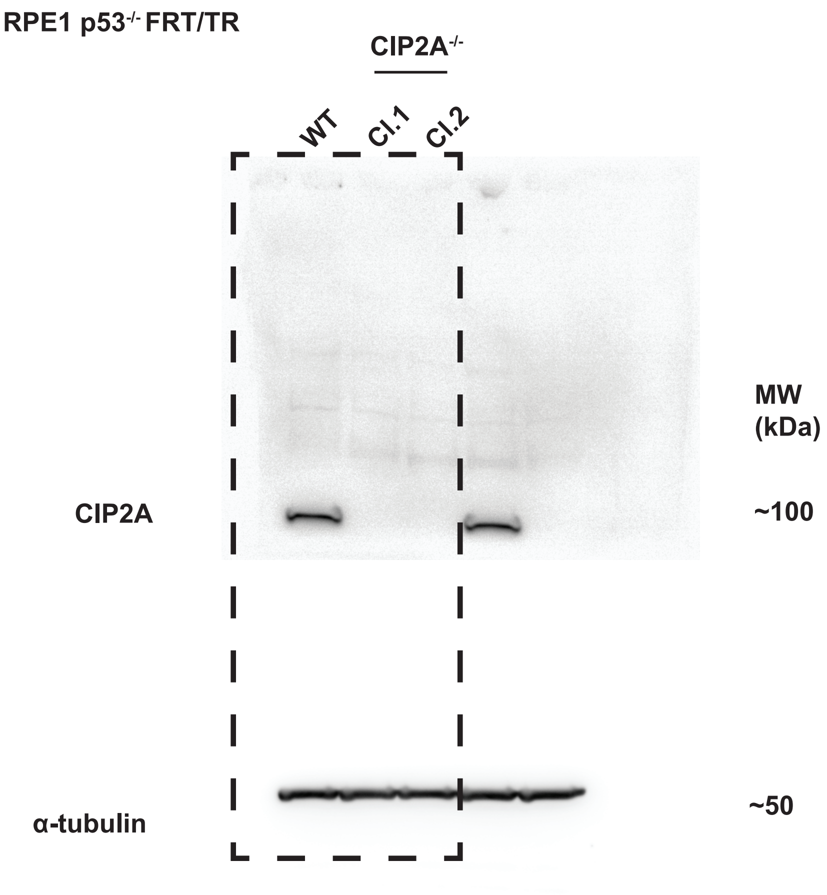

Supplementary Figure 13c

Membranes were cut horizontally and pieces were probed independently with antibodies..

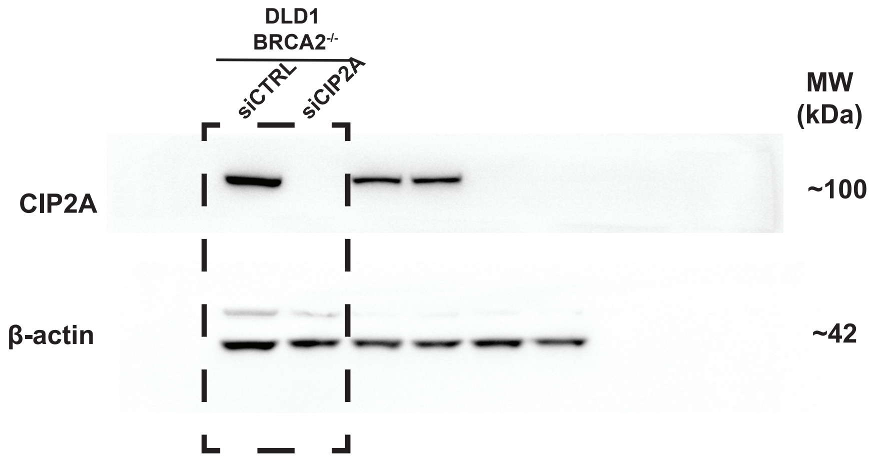

Supplementary Figure 13e

Membranes were cut horizontally and pieces were probed independently with antibodies.
CIP2A was probed initially on the upper piece of the membrane followed by reprobing with anti SLX4.
CIP2A sgnal can still be observed in the lower portion of the SLX4 development below.

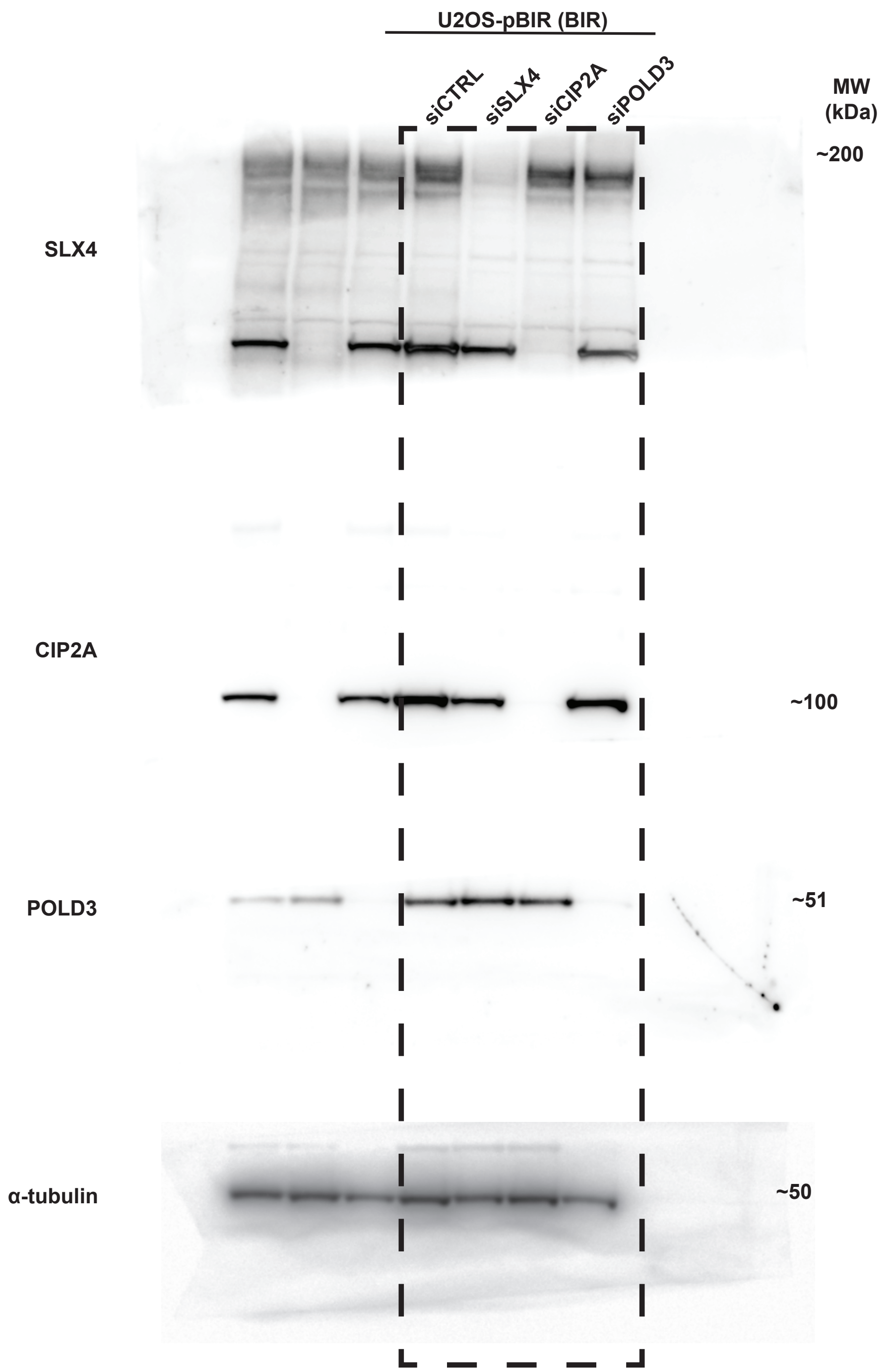

Supplementary Figure 14a

Membranes were cut horizontally and pieces were probed independently with antibodies..

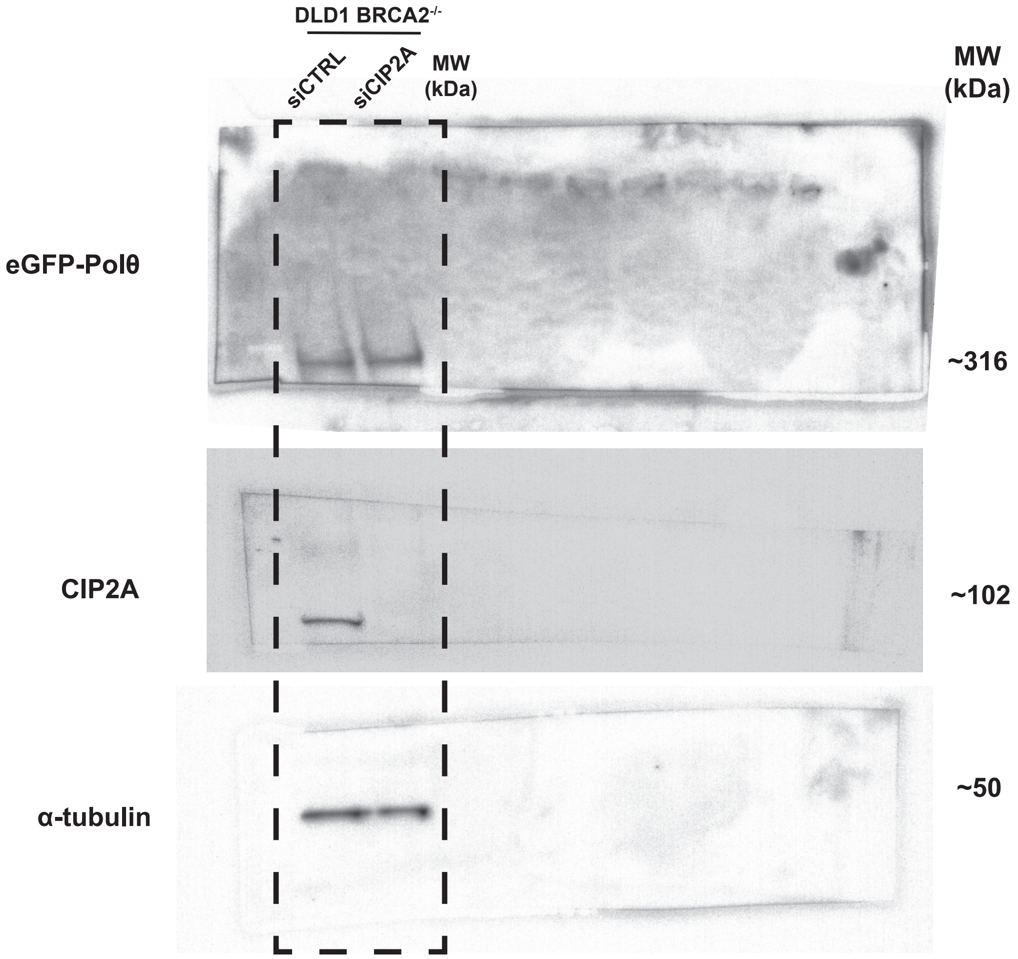

Supplementary Figure 14c

Membranes were cut horizontally and pieces were probed independently with antibodies.

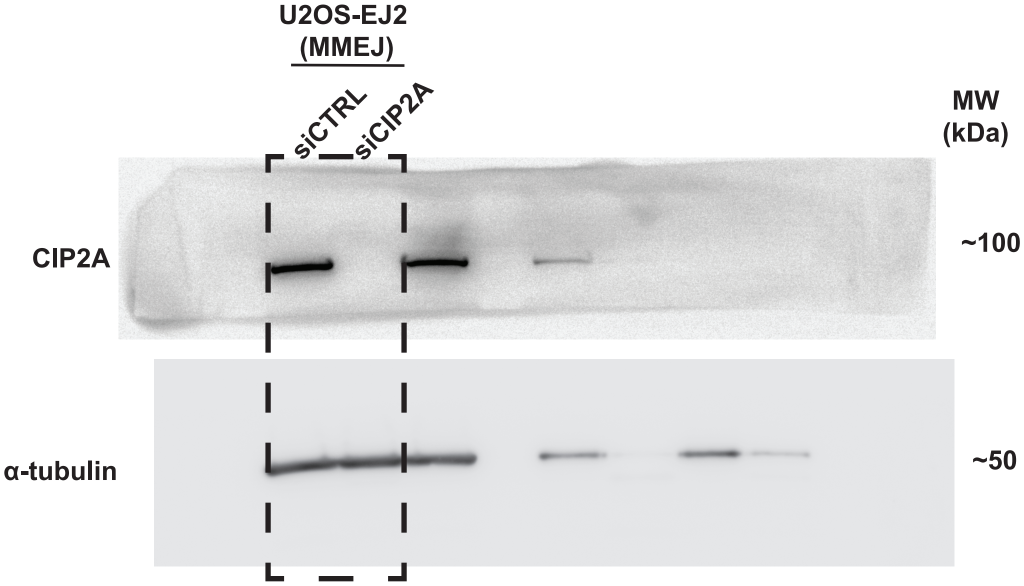

Supplementary Figure 14d

Membranes were cut horizontally and pieces were probed independently with antibodies.

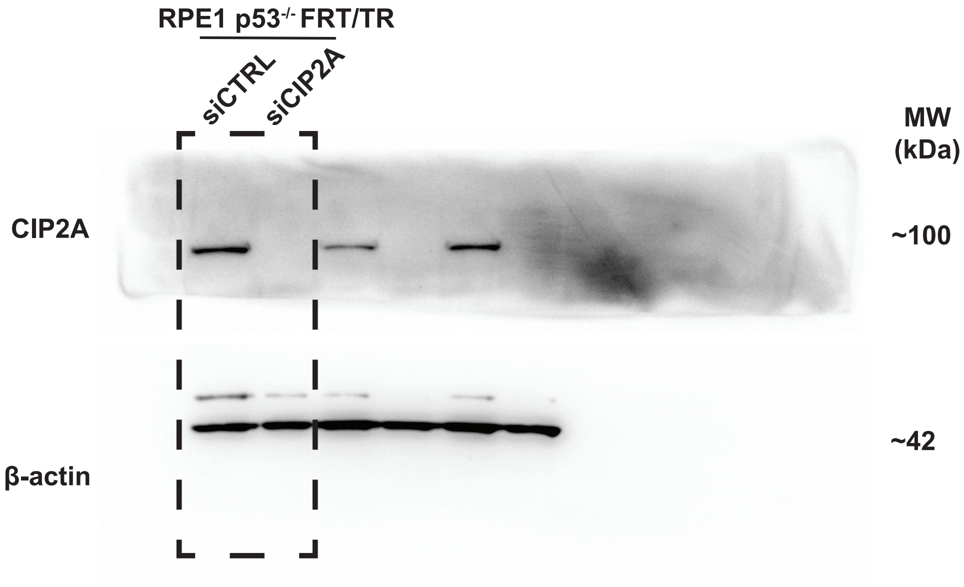

Supplementary Figure 15a

Membranes were cut horizontally and pieces were probed independently with antibodies.

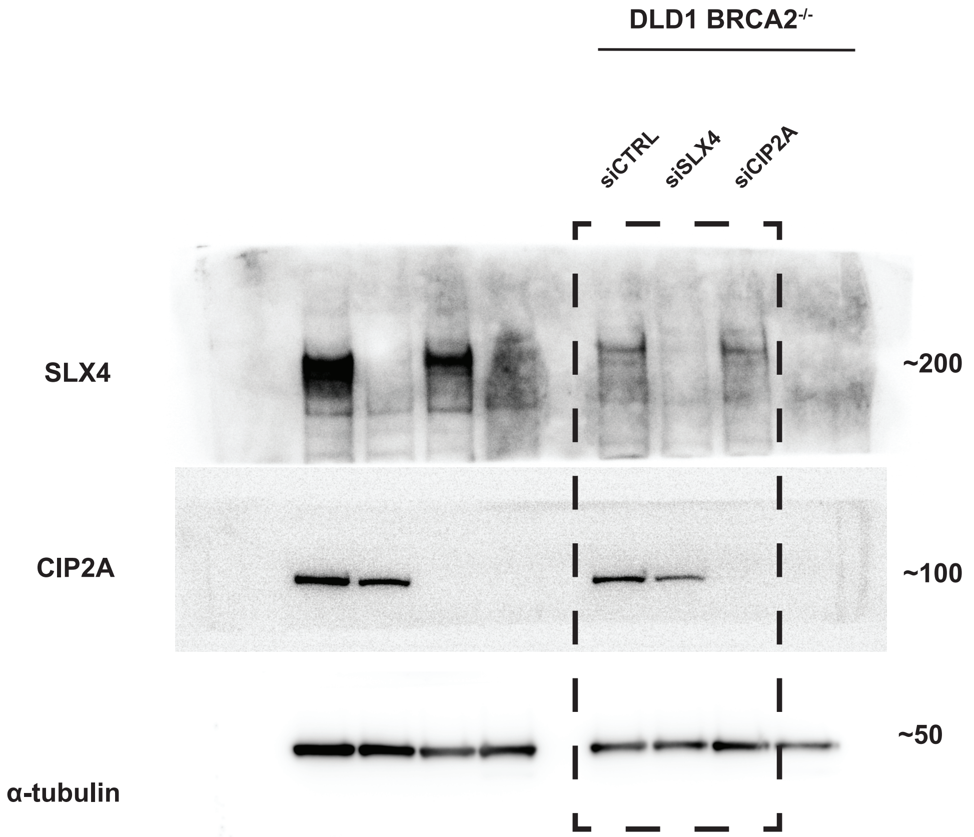

Supplementary Figure 15 c and d
Membranes were cut horizontally to seperate molecular weight of BRCA1 and 2 from MCM2 or Vinculin loading controls. Membrane pieces were incubated in parallel with respective primary antibodies and developed in parallel.

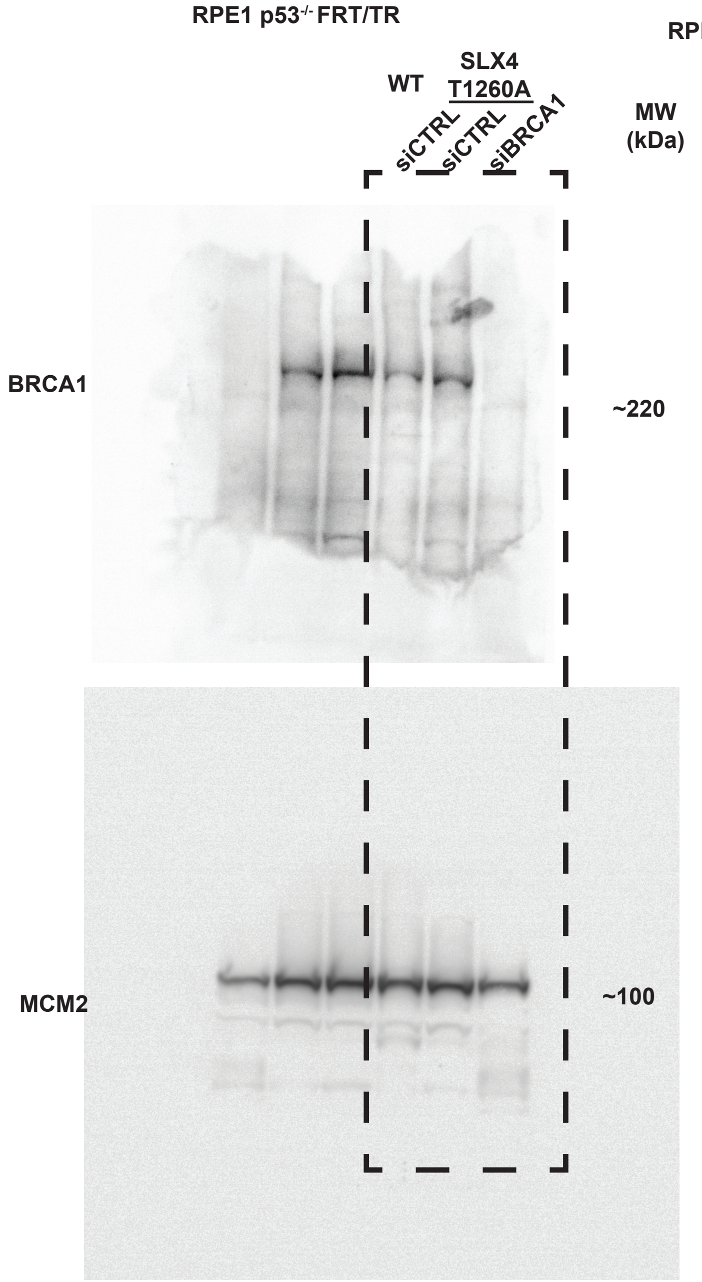

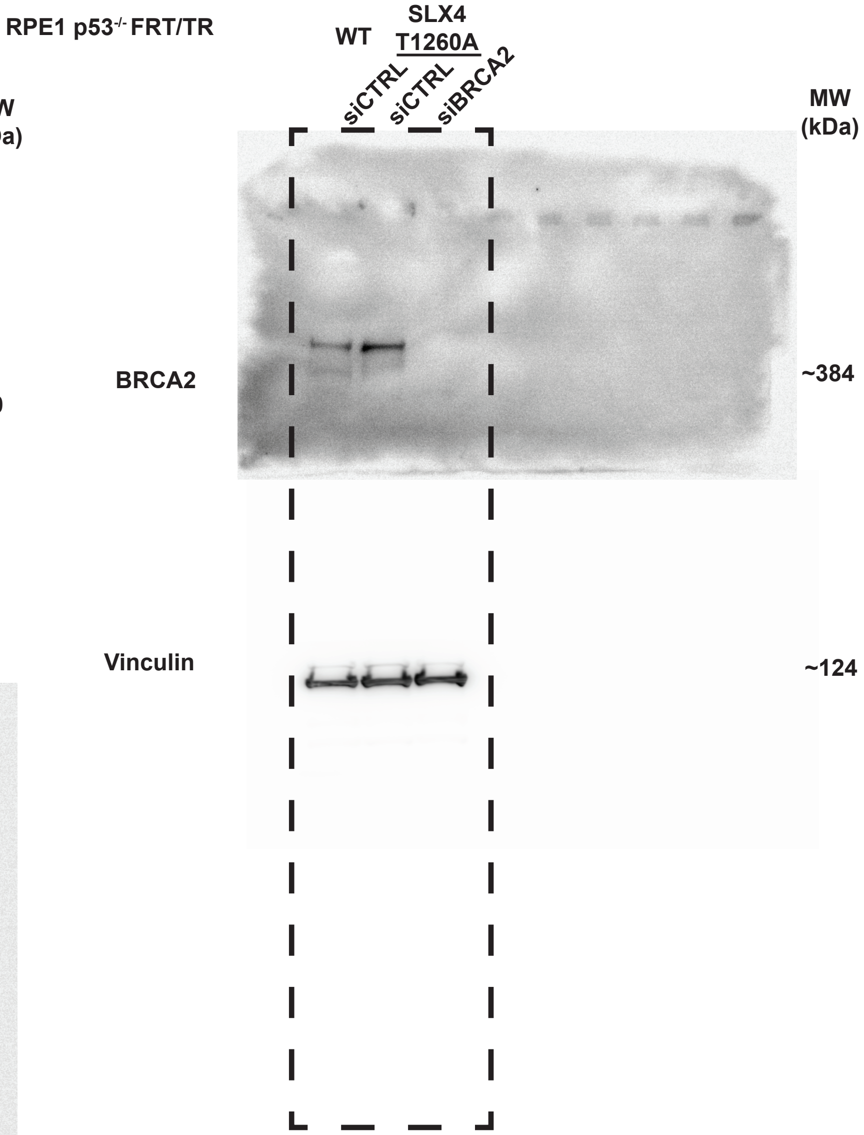

Supplement: Supplementary file 4 — Source Data [file 41467_2025_65594_MOESM4_ESM.zip › Source data/Uncropped western blot images source data file.pdf]
